# Breaking Performance Limits of Zn Anodes in Aqueous Batteries by Tailoring Anion and Cation Additives

**DOI:** 10.1007/s40820-025-01773-6

**Published:** 2025-05-19

**Authors:** Zhaoxu Mai, Yuexing Lin, Jingying Sun, Chenhui Wang, Gongzheng Yang, Chengxin Wang

**Affiliations:** 1 School of Materials Science and Engineering, Sun Yat-Sen (Zhongshan) University, Guangzhou, 510275 People’s Republic of China; 2 State Key Laboratory of Optoelectronic Materials and Technologies, Sun Yat-Sen (Zhongshan) University, Guangzhou, 510275 People’s Republic of China; 3 Instrumental Analysis and Research Center, Sun Yat-Sen (Zhongshan) University, Guangzhou, 510275 People’s Republic of China

**Keywords:** Energy storage, Aqueous zinc-ion batteries, Electrolyte additives, Single Zn(002)-texture, Anion engineering

## Abstract

Crystallographic engineering of Zn anodes to favor the exposure of (002) planes is an effective approach for improving stability in aqueous electrolytes. However, achieving non-epitaxial electrodeposition with a pronounced (002) texture and maintaining this orientation during extended cycling remains challenging. This study questions the prevailing notion that a single (002)-textured Zn anode inherently ensures superior stability, showing that such anodes cannot sustain their texture in ZnSO_4_ electrolytes. We then introduced a novel electrolyte additive, benzyltriethylammonium chloride (TEBAC), which preserves the (002) texture over prolonged cycling. Furthermore, we successfully converted commercial Zn foils into highly crystalline (002)-textured Zn without any pretreatment. Experiments and theoretical calculations revealed that the cationic TEBA^+^ selectively adsorbs onto the anode surface, promoting the exposure of the Zn(002) plane and suppressing dendrite formation. A critical discovery was the pitting corrosion caused by chloride ions from TEBAC, which we mitigated by anion substitution. This modification leads to a remarkable lifespan of 375 days for the Zn||Zn symmetric cells at 1 mA cm^−2^ and 1 mAh cm^−2^. Furthermore, a TEBA^+^-modified Zn||VO_2_ full cell demonstrates high specific capacity and robust cycle stability at 10.0 A g^−1^. These results provide valuable insights and strategies for developing long-life Zn ion batteries.

**Supplementary Information:**

The online version contains supplementary material available at 10.1007/s40820-025-01773-6.

## Highlights

- Cationic benzyltriethylammonium chloride enables high (002)-textured Zn via selective adsorption, outperforming prior additives.
- In situ homogenization converts commercial Zn foil into highly (002)-textured Zn anodes without pretreatments.
- Cl^−^-induced pitting corrosion mechanism uncovered, leading to a breakthrough in performance limits of Zn anodes.

**Supplementary Information:**

The online version contains supplementary material available at 10.1007/s40820-025-01773-6.

## Graphical abstract

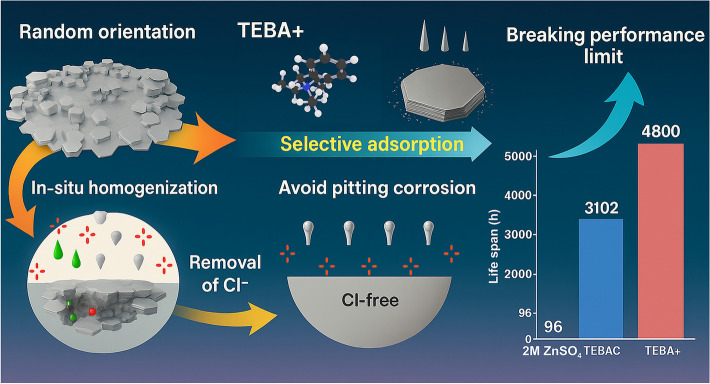

## Introduction

Rechargeable aqueous zinc-ion batteries (ZIBs) have emerged as a promising solution for large-scale energy storage due to their low cost, non-flammability, and high theoretical capacity of 820 mAh g^−1^ [1–4]. However, their practical application is hindered by challenges such as short cycle life and low reversibility, primarily caused by the hydrogen evolution reaction (HER), corrosion, and dendrite growth on Zn anodes [5–7]. HER leads to electrolyte consumption and by-product formation, adversely affecting Zn electrodeposition and resulting in porous Zn [8, 9]. Dendritic growth further increases the risk of short circuits [10, 11], highlighting the need for strategies to enhance the stability and performance of Zn anodes for ZIBs.

To address these challenges, stabilizing Zn metal anodes has become a central focus in ZIBs development. Various strategies have been proposed electrolyte optimization [12–14], crystallographic texture control [15–17], interface engineering [18–20], Zn host structure design [21–23], and separator modification [24–26]. Among these, crystallographic texture control of Zn metal substrates stands out as a practical and effective method to intrinsically improve Zn reversibility. Zn possesses a hexagonal close-packed (HCP) structure, in which the (002) plane is the closed-packed plane. This plane, characterized by minimal surface energy and a compact morphology, is particularly resistant to hydrogen evolution, corrosion, and dendrite formation, making it a desirable orientation for Zn anodes [27–29].

Several methods have been developed to achieve a high (002) texture in Zn anodes, including plastic deformation [30], annealing [31], and electrodeposition [32]. These techniques have successfully produced Zn anodes with highly aligned or even single (002)-textured structures, which effectively suppress side reactions and promote uniform Zn deposition. However, during repeated plating/stripping cycles, the (002) texture tends to degrade, leading to randomly oriented Zn deposition, reduced reversibility, and eventually lead to short circuit. However, most existing studies have primarily focused on fabricating Zn anodes with high (002) texture during initial preparation, with limited attention given to obtaining and maintaining high (002) texture throughout battery cycling, particularly when starting from commercially available Zn foils.

Electrolyte optimization, particularly through the use of specific additives, offers a cost-effective and scalable strategy for controlling Zn electrodeposition and promoting the development of high (002) texture Zn anodes during cycling. For instance, 1-butyl-3-methylimidazolium cation (BMIm^+^) has been reported to selectively adsorb on the (100) and (101) planes, facilitating Zn^2+^ deposition onto the (002) plane through a shielding effect, resulting in a relative texture coefficient (RTC) of 67.26% for the (002) plane after 500 cycles [33]. Similarly, the incorporation of dimethyl(methacryloyloxyethyl) ammonium propane sulfonate (DM) into the electrolyte has enabled highly ordered and compact Zn deposition with an RTC(002) of 50.58% [34]. Additionally, studies utilizing aspartate Zn salt (Zn-Asp) have demonstrated that Zn-Asp preferentially adsorbs on the (101) plane, directing Zn^2+^ deposition along the (002) orientation and achieving an RTC(002) of 76.5% [35]. Despite these advancements, the RTC values reported for the (002) plane remain insufficient to fully exploit the advantages of this crystallographic orientation. Therefore, achieving a highly textured (002) Zn anode and maintaining this texture during practical cycling remains a critical challenge.

In this study, we introduce benzyltriethylammonium chloride (TEBAC) as an electrolyte additive to regulate zinc deposition. TEBAC effectively preserves the (002) texture of Zn anodes with an ultra-high relative texture coefficient (RTC) of 99.8% during extended cycling, preventing the growth of other crystal planes. Furthermore, we demonstrate a facile method to effectively reconstruct and homogenize the texture of commercial Zn foils into a highly crystalline (002)-textured Zn without any pretreatment. Experimental and theoretical analyses reveal that the TEBA^+^ cation selectively adsorbs onto the Zn surface, promoting (002) plane growth and controlling Zn^2+^ nucleation and diffusion, resulting in denser and smoother Zn deposition. In addition, the influence of chloride anions (Cl^−^) from TEBAC on Zn anodes is often under-acknowledged. Our findings reveal that Cl^−^ anions trigger severe pitting corrosion, incite additional hydrogen evolution reactions, and ultimately lead to electrode perforation and battery failure over time. To address this, we replaced Cl^−^ with SO_4_^2−^ in TEBAC, introducing only the TEBA^+^ cation into the ZnSO_4_ electrolyte. This optimized electrolyte, 2 M ZnSO_4_ + 0.04 M TEBA^+^, significantly enhances Zn anode reversibility, achieving a high columbic efficiency of 99.7% and excellent stability in Zn plating/stripping in Zn||Cu cells. The Zn||Zn cells demonstrated a cycle life over 4800 h in coin cells and an impressive over 9000 h (375 days) cycle life in pouch cells at 1 mA cm^−2^ and 1 mAh cm^−2^. Moreover, the Zn||VO_2_ full cell delivered a specific capacity of 267 mAh g^−1^ with an 86.36% capacity retention after 1000 cycles at 10 A g^−1^.

## Experimental Section

### Materials

Zinc sulfate heptahydrate (ZnSO_4_·7H_2_O, ≥ 99.995%), Benzyltriethylammonium hydroxide (TEBAOH, 10% in water), 1-methyl-2-pyrrolidinone (NMP, 99.9%), Oxalic acid dihydrate (H_2_C_2_O_4_, ≥ 99.5%) were purchased from Macklin; Benzyltriethylammonium chloride (TEBAC, 98%), sodium sulfate (Na_2_SO_4_, ≥ 99.0%), zinc chloride (ZnCl_2_, ≥ 99.995%), vanadium pentoxide (V_2_O_5_, ≥ 99.5%), magnesium acetate tetrahydrate (≥ 99%) were purchased from Aladdin. The glass fiber filter (GF/D, GF/A) was purchased from Whatman. All the chemicals were used directly without further purification.

### Preparation of Zn(002) Anodes and VO_2_ Cathodes

#### Preparations of Zn(002) Anodes

The electrodeposition bath consisted of a 1M ZnSO_4_ aqueous solution, prepared by dissolving ZnSO_4_·7H_2_O in deionized water. A straightforward two-electrode electrodeposition method was employed to fabricate the (002)-textured Zn metal electrode. In this setup, commercial Ti foil served as the working electrode and deposition substrate, while commercial Zn foil acted as the counter electrode. The distance between the working and counter electrodes was fixed at 2 cm, and the electrodeposition was carried out using a low-cost 1 M ZnSO_4_ electrolyte under a current density of 100 mA cm^−2^. Vigorous stirring of the electrolyte was maintained throughout the deposition process to ensure uniform ion distribution. To confine Zn deposition to the front side of the Ti substrate, the backside was covered with a polyethylene terephthalate (PET) tape. The electrodeposition process was conducted for 15 min, resulting in the formation of the (002)-textured Zn metal layer.

#### ***Preparations of VO***_***2***_*** Cathodes***

The VO_2_ cathode material was synthesized via a hydrothermal method. Initially, 0.03 mol V_2_O_5_ and 0.09 mol oxalic acid (H_2_C_2_O_4_) were dissolved in 840 mL of deionized water and stirred for 2 h. Subsequently, 0.02 mol magnesium acetate was added to the solution and stirred until fully dissolved. The resulting mixture was transferred to a 1000 mL polytetrafluoroethylene-lined autoclave and maintained at 160 °C for 72 h. After the reaction, the products were collected, washed thoroughly with deionized water, and dried at 60 °C for 12 h to obtain VO_2_ powder. The cathode was prepared by mixing VO_2_ powder, carbon black, and polyvinylidene fluoride (PVDF) in a mass ratio of 7:2:1 using N-methyl-2-pyrrolidone (NMP) as the solvent. The resulting slurry was uniformly coated onto graphite paper and dried at 60 °C for 12 h. The dried cathodes were then punched into disks with a diameter of 12 mm, achieving a VO_2_ mass loading of approximately 2.1 mg per disk (1.85 mg cm^−2^).

### Characterizations

X-ray diffraction (XRD) patterns were recorded using a Rigaku diffractometer with Cu K_*α*_ radiation (*λ* = 0.154 nm). Scanning electron microscopy (SEM) images were acquired using a HITACHI Regulus 8230 scanning electron microscope. Fourier transform infrared (FTIR) spectra were measured with a NICOLET 6700 spectrometer. Raman spectra were obtained using a Renishaw inVia Qontor confocal micro-Raman spectrometer. In situ optical images were captured with an Mshot MJ42 optical microscope.

### Electrochemical Measurements

The electrochemical performance of the electrolytes was evaluated using CR2032 coin cells. Aqueous electrolytes comprising 2 M ZnSO_4_ and 2 M ZnSO_4_ with varying concentrations of TEBAC were used, with glass fiber filters serving as separators. The substitution of Cl^−^ with SO_4_^2−^ in TEBAC was achieved by neutralizing TEBAOH with H_2_SO_4_, followed by the addition of the resulting solution to the ZnSO_4_ electrolyte. Zn^2+^ plating/stripping coulombic efficiency (CE) was measured using asymmetric Cu||Zn cells, while the cycling stability was assessed with symmetric Zn||Zn cells. Galvanostatic cycling and rate capability tests were conducted using a Neware battery testing system. Electrochemical analyses, including linear sweep voltammetry (LSV), electrochemical impedance spectroscopy (EIS, frequency range: 100 kHz to 0.01 Hz, voltage amplitude: 5 mV), cyclic voltammetry (CV), chronoamperometry (CA), and Tafel plots, were performed using a DH7000 electrochemical workstation. The LSV curves were measured from 0 to − 1.5 (V vs. Ag/AgCl), the Tafel curves were measured from − 1.2 to − 0.7 (V vs. Ag/AgCl) at a scan rate of 1 mV s^−1^, and the CA curves were measured at − 0.1 (V vs. Zn/Zn^2+^) for 600 s.

### Density Functional Theory Calculations

Density functional theory (DFT) calculations were conducted to simulate the chemical interactions during the reactions, employing the Vienna Ab-initio simulation package (VASP) [36]. The Perdew–Burke–Ernzerhof (PBE) functional within the framework of the generalized gradient approximation (GGA) was used to compute the exchange–correlation energy, while the projector-augmented wave (PAW) method was adopted for electron–ion interactions [37–39]. To account for van der Waals (vdW) interactions, the DFT-D3 method was incorporated [40]. A plane-wave cutoff energy of 520 eV was applied, and structural visualizations were prepared using Visual Molecular Dynamics (VMD) [41]. Bader analysis was also performed, which unfortunately shows no linear correlation with the adsorption energies and more details are provided in Fig. S21 [42].

Periodic slab models were constructed to represent the (002), (100), and (101) surfaces of Zn. The adsorption configurations of TEBA^+^ on these surfaces are depicted in Fig. 3d. The simulation box parameters for each surface were specified as follows: *a* = *b* = 15.99 Å, *α* = *β* = 90°, *γ* = 120° for Zn (002); *a* = 15.99 Å, *b* = 14.84 Å, *α* = *β* = *γ* = 90° for Zn (100); and *a* = 16.86, *b* = 13.32, *α* = *β* = 90°, *γ* = 103.72° for Zn (101). The TEBA^+^@Zn(002), TEBA^+^@Zn(100), and TEBA^+^@Zn(101) models contained 144, 144, and 126 atoms, respectively. To minimize interactions between periodic slabs, a vacuum gap > 15 Å was introduced. During structural relaxation, the bottom layers of the slab were fixed to maintain stability.

The structures as mentioned before were relaxed using a 2 × 2 × 1 Monkhorst–Pack (MP) k-point mesh until the force on each atom was < 0.05 eV Å^−1^. For total energy calculations of the TEBA^+^@Zn systems, a 3 × 3 × 1 MP k-point mesh and an energy convergence criterion of 10^−6^ eV were employed.

The adsorption energy ($E_{{{\text{ads}}}}$) is calculated utilizing the following Eq. (1):

1 $$E_{{{\text{ads}}}} = E_{{{\text{tot}}}} - E_{{{\text{slab}}}} - E_{{{\text{adsorbate}}}}$$

where $E_{{{\text{tot}}}}$, $E_{{{\text{slab}}}}$ and $E_{{{\text{adsorbate}}}}$ refer to the total energies of the TEBA^+^@Zn system, the Zn slab, and the TEBA^+^ molecule, respectively.

The Gibbs free energy can be calculated by $G = E + ZPE - TS$, in which the value of $ZPE - TS$ is tiny. Comparing with surface energy, the surface free energy needs additional much calculation, and even the final result reveals that $G \approx E$. Thus, the surface free energy can be calculated approximately with total energy of systems (*E*), i.e., surface energy (*E*_surf_) [43] by Eq. (2):

2 $$E_{{{\text{surf}}}} = (E_{{{\text{total}}}} - N_{{{\text{Zn}}}} \times E_{{{\text{Zn}}}} )/2A$$

in which $E_{{{\text{total}}}}$ is the energy of total surface system, $N_{{{\text{Zn}}}}$ is the number of surface Zn atoms, $E_{{{\text{Zn}}}}$ is the energy/atom in in the Zn hcp bulk phase, 2A means the area of the upper and lower surface. We constructed 7 layers dual surface structure with primitive cell, in which the middle layer is fixed. The lattice constants is *a* = *b* = 2.564 Å, *c* = 35.585 Å, *α* = *β* = 90°, *γ* = 120° for Zn(002), *a* = 2.564 Å, *b* = 5.195 Å, *c* = 34.063 Å, *α* = *β* = *γ* = 90° for Zn(100), *a* = 5.793 Å, *b* = 2.564 Å, *c* = 32.591 Å, *α* = *β* = 90°, *γ* = 102.785° for Zn(101). As shown in Fig. S22, the surface energy of different bare Zn surface are illustrated, which suggests that the Zn(002) have the relative lowest surface energy of 0.056 eV Å^−2^ indicating that the most stable surface is (002), as for (100) and (101) surfaces, the surface energy is 0.085 and 0.087 eVÅ^−2^, respectively, revealing relative more active properties. The more active the surface is, the stronger the adsorption for molecule, i.e., the adsorption energy is more negative which is consistent with the result in Fig. 3c.

## Results and Discussion

### Electrolyte-Induced Degradation of Zn(002) Texture

**Fig. 1:**
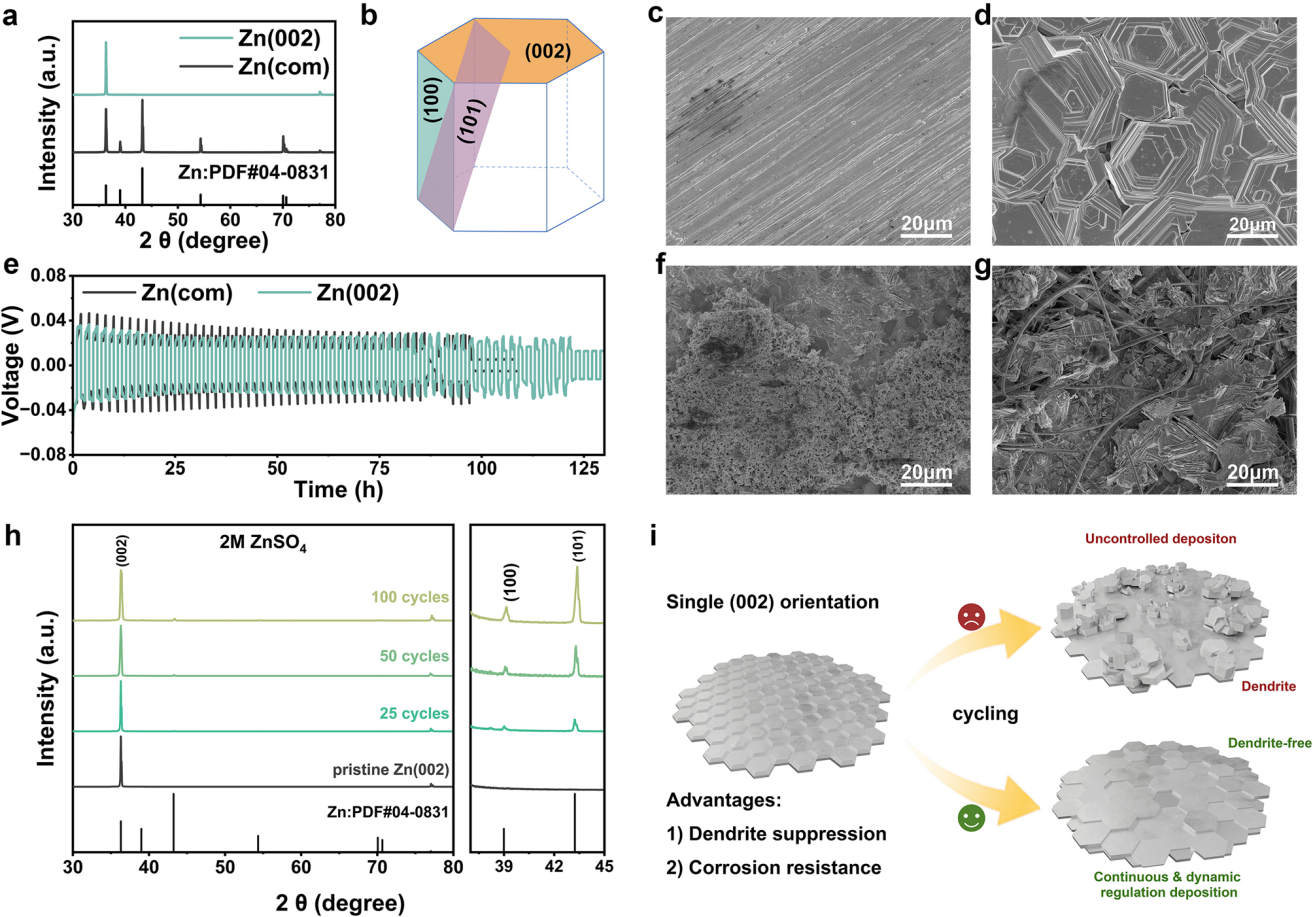
Texture characterization and electrochemical performance of commercial Zn and (002)-textured Zn. **a** XRD patterns of commercial Zn and (002)-textured Zn. **b** Illustration of the hexagonal close-packed (hcp) structure of Zn. **c** SEM images of pristine commercial Zn. **d** SEM images of (002)-textured Zn. **e** Cycling performance comparison between commercial Zn and (002)-textured Zn in 2M ZnSO_4_ electrolyte. **f** SEM images of commercial Zn and **g** SEM images of (002)-textured Zn after 25 cycles. **h** XRD patterns of (002)-textured Zn after various cycles. **i** Schematic diagram of the failure mechanism of (002)-textured Zn and corresponding mitigation strategy

Figure 1b illustrates the hexagonal close-packed (hcp) structure of metallic Zn, where the basal plane aligns with the (002) crystallographic orientation. Zn metal anodes were fabricated using a scalable two-electrode electrodeposition technique. Commercial Ti foil served as the working electrode and deposition substrate, while commercial Zn foil was used as the counter electrode. The electrolyte consisted of an aqueous 1 M ZnSO_4_ solution, which was vigorously stirred throughout the electrodeposition process. Deposition was conducted at a current density of 100 mA cm^−2^ with a cutoff capacity of 25 mAh cm^−2^. The resulting Zn layer could be easily detached from the Ti substrate, forming a free-standing Zn foil (Fig. S1) with a predominant (002) texture (denoted as Zn(002)), as confirmed by XRD (Fig. 1a). Figure 1c, d shows the SEM images of commercial Zn foil and the as-prepared Zn(002) foil, respectively. The electrodeposited Zn foil displays a smooth, horizontally aligned hexagonal surface, highlighting the highly exposed (002) texture (Figs. 1d and S2).

Figure 1e presents the cycling stability of symmetric Zn||Zn coin cells with both commercial Zn (denoted as Zn(com)) and Zn(002) anodes, tested in a 2 M ZnSO_4_ aqueous electrolyte at a current density of 1 mA cm^−2^ and a areal capacity of 1 mAh cm^−2^. Both cell types exhibited limited stability, with Zn(002) cells failing after 121 h, showing no significant improvement over Zn(com) cells, which failed after 97 h. To investigate these results, we examined the surface morphology of both anodes after 25 cycles using scanning electron microscopy (SEM). The Zn(com) electrode developed a rough, moss-like surface (Fig. 1f), while the Zn(002) electrode, initially smooth with horizontally aligned hexagonal features, showed significant deformation, resulting in an irregular surface topography (Fig. 1g). The XRD patterns of the Zn(002) anode at different cycling stages (Figs. 1h and S3) revealed that the (002) orientation could not be maintained in the 2 M ZnSO_4_ electrolyte. As cycling progressed, diffraction peaks corresponding to other crystal planes emerged and intensified, indicating a loss of the (002) texture. Figure 1i schematically illustrates the failure of the Zn(002) anode to retain its single (002) texture after extended cycling in conventional ZnSO₄ electrolyte. To achieve a dendrite-free Zn anode with long-term cycling stability, it is crucial to dynamically regulate Zn deposition to preserve the (002) texture.

### TEBAC Stabilized and Enhanced (002) Texture for Long-term Cycling

**Fig. 2:**
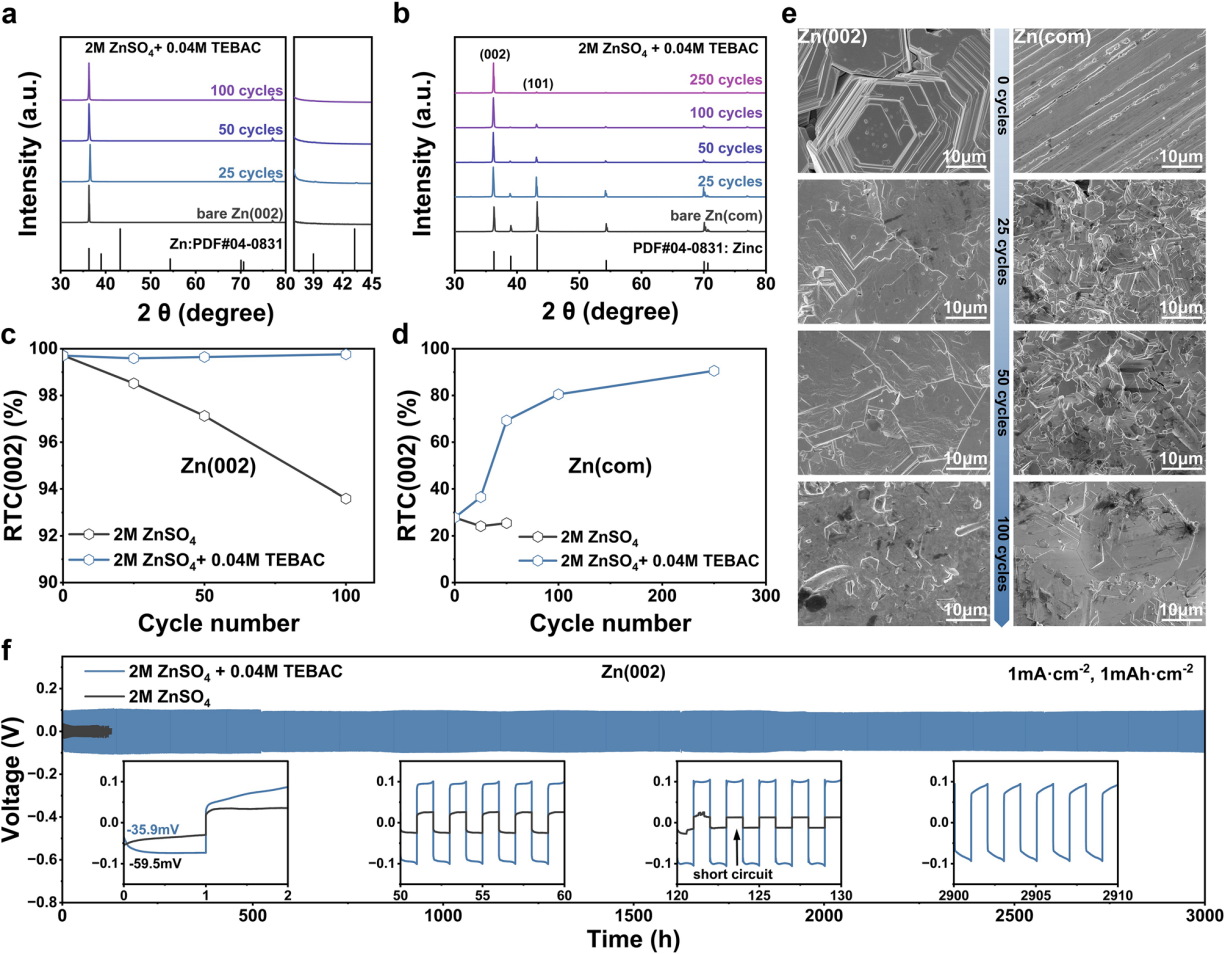
Texture and morphology evolution of commercial Zn and (002)-textured Zn in after the addition of TEBAC. **a** XRD patterns of (002)-textured Zn cycled in 2 M ZnSO_4_ + 0.04 M TEBAC electrolyte at different cycle numbers. **b** XRD patterns of commercial Zn cycled in 2 M ZnSO_4_ + 0.04 M TEBAC electrolyte at different cycle numbers. **c** RTC (002) values of (002)-textured Zn. **d** RTC (002) values of commercial Zn at varying cycle numbers in 2 M ZnSO_4_ + 0.04 M TEBAC electrolyte. **e** SEM images showing the surface morphology of (002)-textured Zn and commercial Zn after different cycle numbers in 2 M ZnSO_4_ + 0.04 M TEBAC electrolyte. **f** Cycling performance comparison of (002)-textured Zn in various electrolytes

To control the Zn electrodeposition process, TEBAC was incorporated into a 2 M ZnSO_4_ electrolyte, with an optimal concentration of 0.04 M identified, as shown in Figs. S4 and S5. This formulation effectively preserved the (002) texture of the Zn(002) anodes throughout electrochemical cycling, as demonstrated in Figs. 2a and S6. To further investigate the role of TEBAC in maintaining the (002) texture, Zn||Zn symmetric cells were assembled using Zn(com) anodes. In the 2 M ZnSO_4_ electrolyte, the intensity of the (002) peak for the Zn(com) anodes decreased after 25 cycles (Fig. S7). In contrast, with the addition of TEBAC, the intensity of the (002) peak increased over cycling and became the dominant diffraction peak, surpassing the (101) peak only after 25 cycles. Simultaneously, diffraction peaks corresponding to non-(002) crystal planes diminished with increasing cycles, indicating enhanced texture stability (Fig. 2b). After 250 cycles, a highly crystalline (002)-textured Zn was obtained.

The evolution of the Zn texture was quantitatively analyzed by calculating the relative texture coefficient (RTC) for each crystal plane using the following Eq. (3) [44]:

3 $$RTC_{{\left( {hkl} \right)}} = \frac{{I_{{\left( {hkl} \right)}} /I_{{0\left( {hkl} \right)}} }}{{\sum \left[ {I_{{\left( {hkl} \right)}} /I_{{0\left( {hkl} \right)}} } \right]}} \times 100\%$$

where, $I_{{\left( {hkl} \right)}}$ represents the intensity from the experimental XRD patterns, and $I_{{0\left( {hkl} \right)}}$ is the intensity of the standard Zn sample (JCPDF no.04-0831). Figure 2c demonstrates the evolution of the relative texture coefficient (RTC) for the (002) texture in Zn anodes under different electrolyte conditions. For Zn(002) anodes in 2 M ZnSO_4_ electrolyte, the RTC for the (002) plane gradually declines to 93.6% after 100 cycles. In contrast, when TEBAC is introduced, the RTC remains exceptionally stable, maintaining a high value of 99.8% after 100 cycles. Similarly, for Zn(com) anodes, the RTC of the (002) plane shows a significant improvement, increasing from an initial value of 29.0–80.5% after 100 cycles in the TEBAC-containing electrolyte. Notably, this value further rises to 90.6% after 250 cycles, indicating a progressive enhancement in the (002) texture facilitated by TEBAC (Fig. 2d).

The morphological evolution of both Zn(002) and Zn(com) anodes during cycling was observed via SEM (Fig. 2e). On Zn(002) anodes, the horizontally aligned Zn platelets undergo lateral growth during the plating/stripping process, forming a densely packed, enlarged hexagonal crystalline structure. Similarly, Zn(com) anodes develop an array of horizontally aligned Zn platelets, which expand as cycling progresses. The inclusion of TEBAC facilitates the dynamic and continuous modulation of Zn deposition, thereby preserving and even enhancing the (002) texture. This enhancement significantly extends the operational lifespan of Zn(002) anodes, achieving an impressive 3000 h—approximately a 24-fold increase compared to the 121 h observed with the ZnSO_4_ electrolyte alone (Fig. 2f).

### Mechanistic Insight into TEBAC-Regulated Homogeneous Zn Deposition

**Fig. 3:**
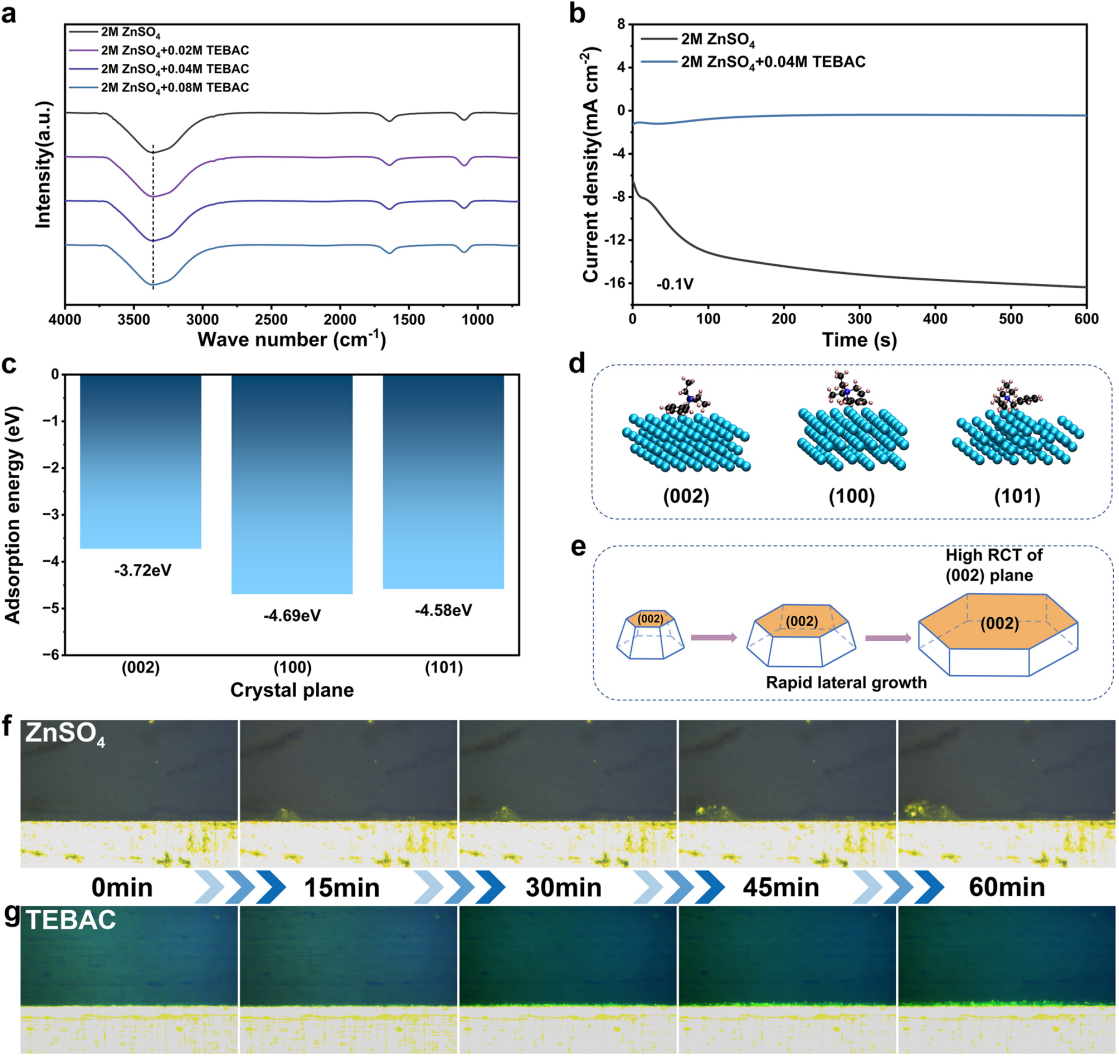
**a** FTIR spectra of 2 M ZnSO_4_ electrolyte with different concentration of TEBAC. **b** Chronoamperometry (CA) curves measured using Zn||Zn cells in 2 M ZnSO_4_ electrolytes, with and without TEBAC, at a constant potential of − 0.1 V. **c** Adsorption energy of TEBA^+^ on (002), (100), and (101) crystal planes. **d** Adsorption configurations of TEBA^+^ on (002), (100) and (101) crystal planes. **e** Schematic diagram illustrating the lateral growth of the (002) crystal plane. In-suit optical observation of Zn deposition in **f** 2 M ZnSO_4_ electrolyte, **g** 2 M ZnSO_4_ + 0.04 M TEBAC electrolyte

To investigate the impact of the TEBAC additive on the solvation structure surrounding Zn^2+^ ions, Fourier transform infrared (FTIR) spectroscopy and Raman spectroscopy were employed. Fig. 3a and S8 present the spectra of 2 M ZnSO_4_ electrolytes with varying TEBAC concentrations. Notably, no significant spectral changes were observed, indicating that TEBAC does not change the solvation structure of Zn^2+^.

DFT calculations were performed to explore the preferential growth mechanism of the (002) plane induced by TEBAC, specifically focusing on the adsorption energies between the TEBA^+^ cation and different Zn crystal planes. The (002), (100), and (101) planes were selected for this analysis. Figure 3c shows the side views of the TEBA^+^ adsorption configurations on these planes, alongside the calculated adsorption energies. The adsorption energy of TEBA^+^ on the (002) plane was found to be − 3.72 eV, less negative than that on the (100) (− 4.69 eV) and (101) (− 4.58 eV) planes. This suggests a weaker interaction between TEBA^+^ and the Zn(002) plane, which facilitates Zn^2+^ diffusion along other directions, thereby reducing the deposition rate on the (002) plane. According to Bravais‘ law, this restricted deposition along the [002] direction promotes the exposure of the (002) texture, leading to enhanced lateral Zn growth [45–47], as schematically illustrated in Fig. 3d, e. Additional Bader analysis and surface energy analysis are placed in Supporting Information (Figs. S21 and S22). These computational insights align with experimental observations of texture evolution during cycling. During deposition, TEBA^+^ selectively adsorbs on non-(002) planes (e.g., (100) and (101)), allowing Zn^2+^ to preferentially deposit and laterally grow on the (002) plane. Concurrently, during stripping, non-(002) planes with higher surface energy (as confirmed by surface energy analysis in Fig. S22) are more readily dissolved [18], leaving the lower-energy (002) planes relatively intact. This dynamic interplay drives the gradual transformation of randomly oriented Zn(com) anodes into highly (002)-textured structures during cycling (Fig. 2b, d, and e). Even under repeated plating/stripping stresses, TEBA^+^ ensures continuous self-reinforcement of the (002) textured, enabling the Zn(com) anode to achieve a progressive increase in (002) RTC from 29.0% to 90.6% over 250 cycles (Fig. 2d).

The chronoamperometry (CA) technique was employed to examine Zn nucleation in different electrolytes, with tests conducted at a potential of − 0.1 V for Zn||Zn cells. As shown in Fig. 3b, in the pristine ZnSO_4_ electrolyte, the current response increased steadily over 600 s. In contrast, the electrolyte containing TEBA⁺ exhibited a rapid stabilization of the current, indicating a more efficient and uniform Zn nucleation process, likely due to the effective adsorption of TEBA^+^ [48]. In situ optical microscopy was utilized to observe the electroplating behavior of Zn^2+^ ions, with the morphology evolution of the Zn electrode surface monitored at a constant deposition current density of 2 mA cm^−2^. Figure 3f reveals that in the pristine ZnSO_4_ electrolyte, the zinc electrodes developed uneven protrusions after 15 min, which became more pronounced with extended plating times. In contrast, Zn electrodes in the TEBAC-containing electrolyte maintained a smooth and compact surface throughout the plating process (Fig. 3g).

### TEBAC Enhances Interfacial Stability and Ion Kinetics

**Fig. 4:**
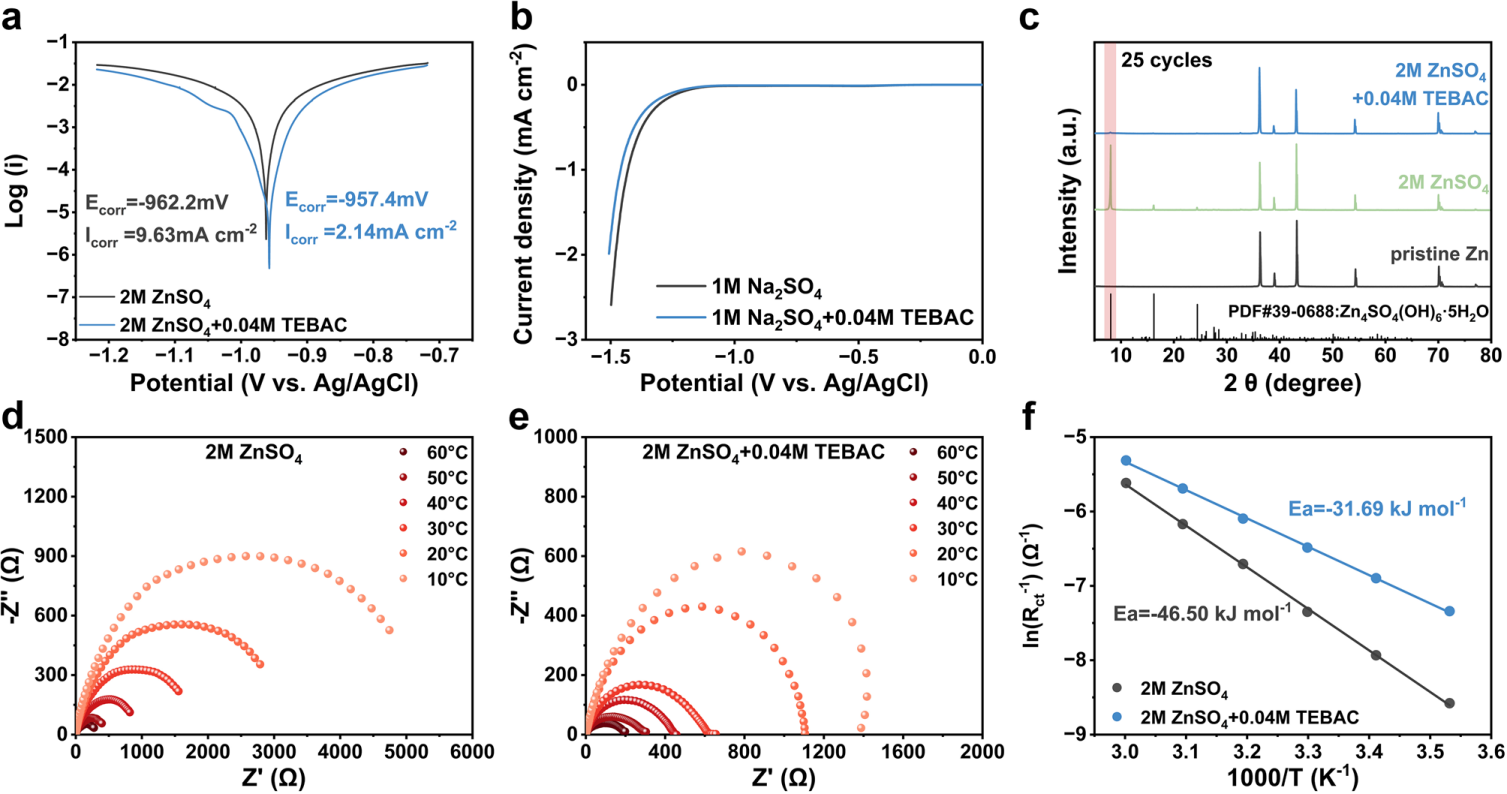
**a** Tafel plots comparing Zn anodes in electrolytes with and without the TEBAC additive. **b** LSV curves for hydrogen evolution reaction (HER) in various electrolytes. **c** XRD patterns of Zn anodes after 25 cycles in different electrolytes. Electrochemical impedance spectroscopy (EIS) results at different temperatures for **d** 2 M ZnSO_4_ and **e** 2 M ZnSO_4_ + 0.04 M TEBAC. **f** Arrhenius curves and the fitted desolvation activation energies

The anti-corrosion efficacy of TEBAC on Zn anodes was evaluated using Tafel plot analysis. As shown in Fig. 4a, Zn anodes in the TEBAC-containing electrolyte exhibited a lower corrosion current density (*I*_corr_) and a minor positive shift in corrosion potential (*E*_corr_) compared to those in the pristine ZnSO₄ electrolyte. This indicates that TEBAC effectively hinders the spontaneous corrosion of the zinc anode [49, 50]. To assess TEBAC’s impact on hydrogen evolution, LSV was performed. Given the proximity of the Zn^2^⁺ reduction potential to that of H₂O reduction, the influence of TEBAC on the HER was studied in Na₂SO₄ electrolytes, as shown in Fig. 4b. A significant suppression of HER was observed, likely due to the reduced activity of water in the presence of TEBAC. Specifically, the hydrophobic groups-benzyl of TEBA^+^ cation self-assemble at the electrolyte/Zn interface via hydrophobic interactions and electrostatic attraction, forming a stable hydrophobic layer. This layer limits the accessibility of water molecules to active sites while maintaining sufficient Zn^2^⁺ permeability [51]. The enhanced corrosion resistance provided by TEBAC was further confirmed through XRD analysis of Zn anodes cycled in different electrolytes. Figure 4c demonstrates that after 25 cycles, the Zn anode in the pristine electrolyte showed pronounced diffraction peaks corresponding to the typical corrosion by-product Zn₄SO₄(OH)₆·5H₂O. In contrast, the anode cycled in the TEBAC-containing electrolyte exhibited minimal peaks associated with this corrosion product.

To evaluate the impact of the electrolyte additive on zinc plating/stripping kinetics, electrochemical impedance spectroscopy (EIS) was performed on Zn||Zn symmetric cells at temperatures ranging from 10 to 60 °C, as shown in Fig. 4d, e. The desolvation of Zn^2+^ at the electrode–electrolyte interface encounters significant resistance due to the strong Coulombic interactions between divalent ions and their solvation shell. This resistance can lead to side reactions, such as hydrogen evolution, making desolvation a rate-limiting step for Zn^2+^ deposition. The desolvation process can be described by the activation energy (*E*ₐ), which is determined using the Arrhenius Eq.: $\frac{1}{{R_{ct} }} = Aexp\left( { - \frac{{E_{a} }}{RT}} \right)$, where $R_{ct}$ is the charge transfer resistance, $A$ is the pre-exponential factor, $R$ is the gas constant, and $T$ is the absolute temperature. As shown in Fig. 4f, the activation energy for desolvation in the TEBAC-containing electrolyte is significantly lower at − 31.69 kJ mol^−1^, compared to − 46.50 kJ mol^−1^ for the pristine ZnSO_4_ electrolyte. This indicates that TEBAC facilitates the removal of the Zn^2+^ solvation shell, thereby enhancing ion transfer kinetics [52, 53].

### Remove Cl⁻ to Suppress Pitting Corrosion and Extends Anode Stability

**Fig. 5:**
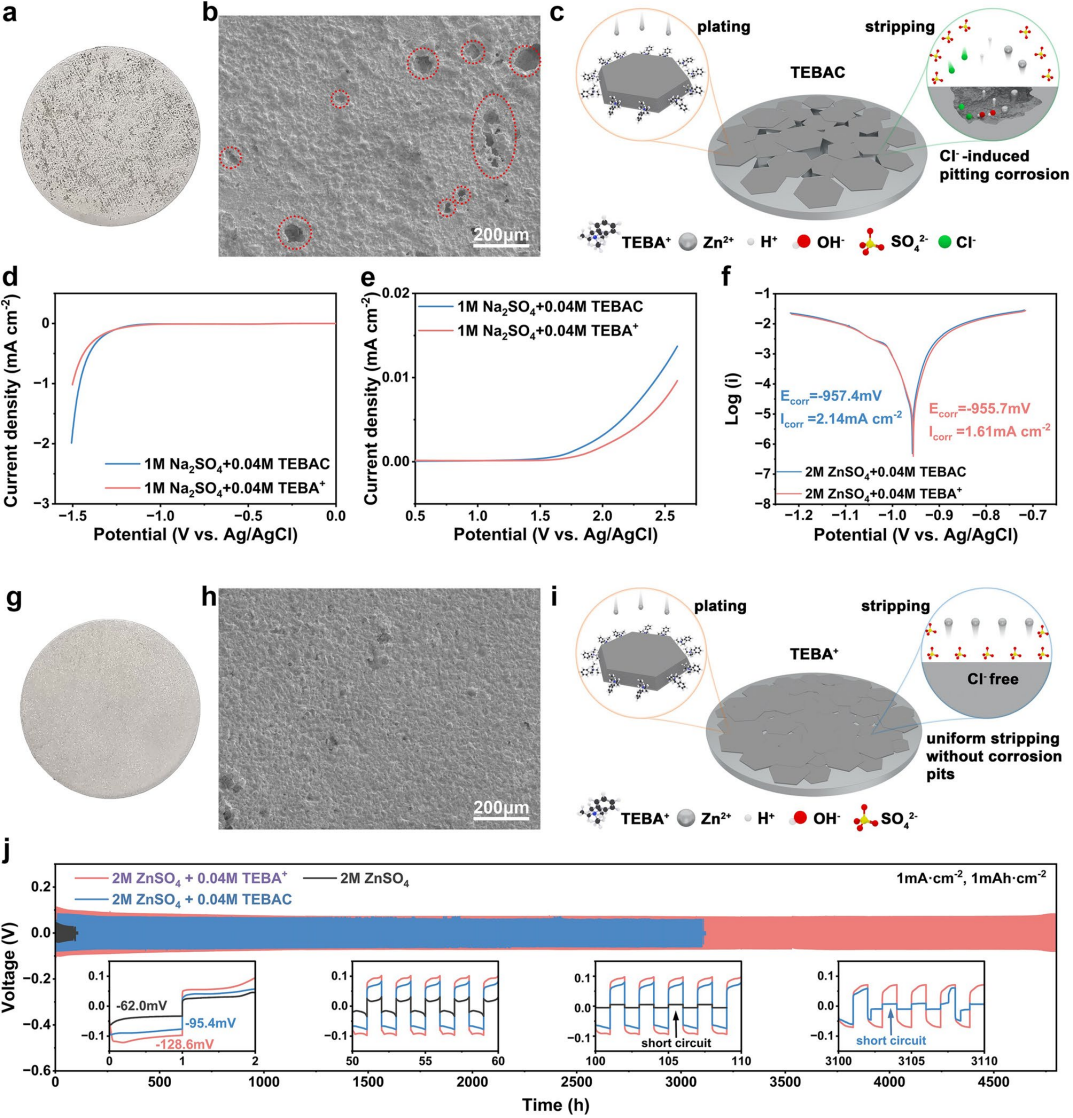
**a** Digital image of the Zn anode after 100 cycles in 2 M ZnSO_4_ + 0.04 M TEBAC electrolyte. **b** SEM images of the Zn anode after 100 cycles in 2 M ZnSO_4_ + 0.04 M TEBAC. **c** Schematic diagram of Cl^−^-induced pitting corrosion. LSV curves for **d** HER and **e** OER in various electrolytes. **f** Tafel plots comparing Zn anodes in electrolytes with and without Cl^−^. **g** Digital image of the Zn anode after 100 cycles in 2 M ZnSO_4_ + 0.04 M TEBA^+^ electrolyte. **h** SEM images of the Zn anode after 100 cycles in 2 M ZnSO_4_ + 0.04 M TEBA^+^. **i** Schematic diagram of uniform stripping in the absence of Cl^−^. **j** Comparison of cycling performance of commercial Zn anodes in different electrolytes

The introduction of TEBAC successfully enables dendrite-free Zn deposition; however, black spots were observed on the cycled Zn anode, as shown in Fig. 5a. SEM images in Fig. 5b reveal these spots as pits, indicative of pitting corrosion, a common form of corrosion for Zn metal in aqueous environments [54]. To investigate the origin of this corrosion, ZnCl₂ was added to a 2 M ZnSO₄ electrolyte. The Zn||Zn cells with this electrolyte showed no performance improvement (Fig. S9), and similar pits appeared on Zn anodes after the addition of Cl^−^ ions (Fig. S10), suggesting that Cl^−^ triggers pitting corrosion. Further electrochemical analysis (Fig. S11) shows an increase in I_corr_ and a more negative E_corr_ with the addition of Cl^−^, indicating an increased corrosion tendency. It is hypothesized that, during stripping, Cl^−^ ions accumulate at surface defects or impurities, forming localized corrosion cells and inducing pitting [55], as illustrated in Fig. 5c.

To mitigate Cl^−^-induced pitting corrosion, Cl^−^ was replaced with SO_4_^2−^ in the TEBAC-containing electrolyte, introducing only the cationic TEBA^+^. The removal of Cl^−^ led to a reduced HER potential and an increased oxygen evolution reaction (OER) potential, as shown in the LSV curve in Fig. 5d, e. The tafel plot in Fig. 5f further confirms a reduction in I_corr_ from 1.61 mA cm⁻^2^ and an increase in E_corr_ to − 955.7 mV, highlighting the beneficial effect of Cl^−^ absence on the electrochemical process. As a result, corrosion pits on cycled Zn anodes were significantly reduced in the 2 M ZnSO_4_ + 0.04 M TEBA^+^ electrolyte (Figs. 5g, h and S12). The absence of Cl^−^ ions promotes uniform stripping and enhances Zn anode reversibility, leading to an extended lifespan of 4800 h at 1 mA cm^−2^ and 1 mAh cm^−2^, far surpassing the 3100-h lifespan observed in Cl^−^-containing electrolytes (Fig. 5j).

### TEBA^+^ Enables Ultra-Stable and High-Efficiency Zn Battery Cycling

**Fig. 6:**
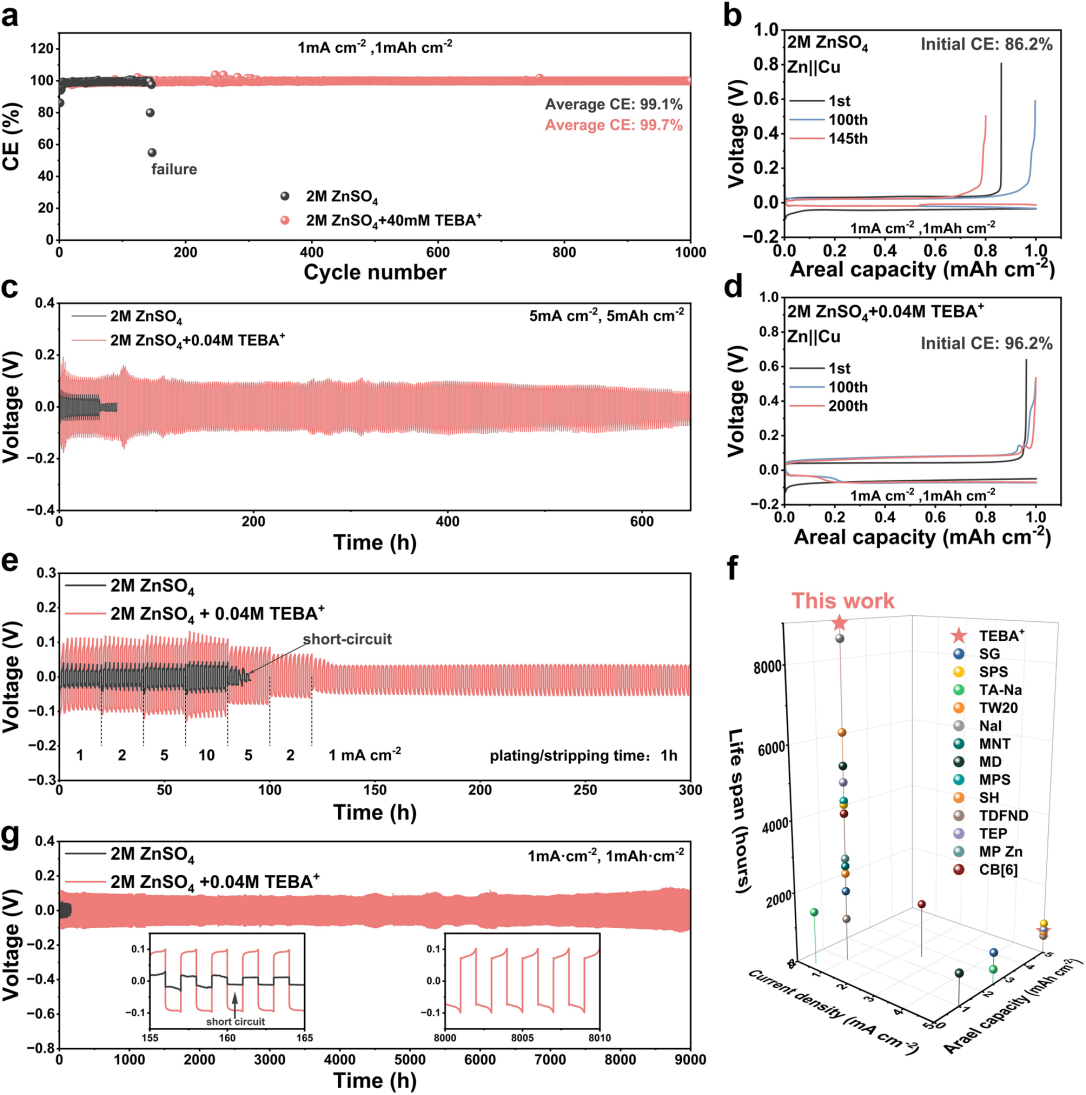
Electrochemical performance of the TEBA^+^ electrolytes. **a** Coulombic efficiency (CE) of Zn||Cu cells with a cutoff charging voltage of 0.5 V at 1 mA cm^−2^ and 1 mAh cm^−2^. Voltage-capacity curves of Zn||Cu cells in **b** 2 M ZnSO_4_ and **d** 2 M ZnSO_4_ + 0.04 M TEBA^+^ electrolytes. **c** Cycling performance of Zn||Zn cells with and without TEBA^+^ at 5 mA cm^−2^ and 5 mAh cm^−2^. **e** Rate performance of Zn||Zn cells with and without TEBA^+^. **f** Comparison of cycling stability of Zn||Zn cells in this study versus other recently reported works. **g** Cycling performance of Zn||Zn pouch cells with and without TEBA^+^

Zn||Cu asymmetric cells were assembled to evaluate the reversibility of Zn plating/stripping in ZnSO_4_ electrolyte. The cell with plain ZnSO_4_ exhibited an initial coulombic efficiency (CE) of 86.2%, which rapidly decreased after 145 cycles, primarily due to short-circuiting caused by dendrite formation (Fig. 6b). In contrast, the Zn||Cu cell using the ZnSO_4_ + TEBA^+^ electrolyte started with a higher CE of 96.2% (Fig. 6d) and maintained stable performance for over 1000 cycles, achieving an average CE of 99.7% (Fig. 6a). Figure 6c demonstrates the stability of the Zn||Zn cells at a current density of 5 mA cm^−2^ and a capacity of 5 mAh cm^−2^. The Zn||Zn cell with ZnSO_4_ electrolyte short-circuited in just 42 h due to extensive dendrite formation and severe side reactions. In stark contrast, the Zn||Zn cell with ZnSO_4_ + TEBA^+^ electrolyte exhibited exceptional stability, sustaining Zn plating/stripping for over 650 h under identical conditions. At a higher current density of 10 mA cm^−2^ and a capacity of 2 mAh cm^−2^, the Zn||Zn cell with ZnSO_4_ + TEBA^+^ electrolyte sustained continuous cycling for over 1100 h, while the cell with plain ZnSO_4_ electrolyte failed after just 200 h (Fig. S13). Rate performance tests (Fig. 6e) demonstrated the superior stability of the ZnSO_4_ + TEBA^+^ electrolyte across current densities ranging from 1 to 10 mA cm^−2^, whereas the plain ZnSO_4_ electrolyte caused short-circuiting at 10 mA cm^−2^. Furthermore, a Zn||Zn pouch cell utilizing ZnSO_4_ + TEBA^+^ electrolyte achieved an ultra-long lifespan of 9000 h at 1 mA cm^−2^ and 1 mAh cm^−2^ (Fig. 6g), far surpassing most reported systems, as summarized in Fig. 6f [13, 45, 46, 56–65]. These results underscore the effectiveness of TEBA^+^ in significantly enhancing the cycling stability and performance of Zn-based cells.

### TEBA^+^ Enables High-Rate and Long-Cycling Zn||VO₂ Full Cells

**Fig. 7:**
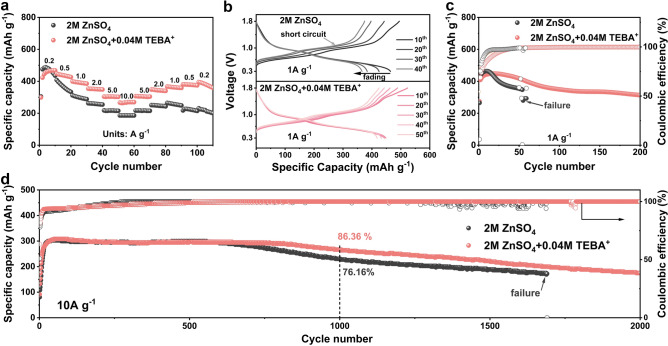
Performance of Zn||VO_2_ cells. **a** Rate performance of Zn||VO_2_ cells in different electrolytes at current densities of 0.2, 0.5, 1.0, 2.0, 5.0, and 10.0 A g^−1^. **b** Voltage profiles of Zn||VO_2_ cells in different electrolytes at current density of 1 A g^−1^. Cycling stability of Zn||VO_2_ cells in different electrolytes at **c** 1 A g^−1^ and **d** 10 A g^−1^

The efficacy of TEBA^+^ additives was validated using Zn||VO_2_ full cells in ZnSO_4_ electrolyte, with and without TEBA^+^. The XRD pattern (Fig. S14) and SEM image (Fig. S17) confirmed the successful synthesis of the VO_2_ cathode. CV curves (Fig. S15) revealed that the introduction of TEBA^+^ slightly increased the voltage separation between reduction and oxidation peaks, attributed to increased internal resistance [66] (Fig. S16). Rate performance tests (Fig. 7a) demonstrated that Zn||VO_2_ cells with ZnSO_4_ + TEBA^+^ electrolyte exhibited superior specific capacities of 457, 436, 392, 351, 303, and 268 mAh g^−1^ at current densities of 0.2, 0.5, 1.0, 5.0, and 10.0 A g^−1^, respectively. These values were consistently higher than those of cells using plain ZnSO_4_ electrolyte, which delivered 469, 366, 298, 258, 216, and 186 mAh g^−1^ at the same current densities. This indicates enhanced Zn^2+^ insertion/extraction properties with TEBA^+^. At 1 A g^−1^, cells without TEBA^+^ suffered a drastic capacity decline from 434 to 279 mAh g^−1^ over 50 cycles, eventually short-circuiting due to dendrite formation (Fig. S18). In contrast, cells with TEBA^+^ maintained a specific capacity of 314 mAh g^−1^ after 200 cycles, achieving a capacity retention of 76.96% (Fig. 7a, b). The Zn anode cycled in with TEBA^+^ demonstrated a highly (002)-textured surface without dendrite formation, which was also confirmed by XRD results (Figs. S19 and S20). Long-term cycling performance at 10 A g^−1^ (Fig. 7d) showed that TEBA^+^ increased capacity retention from 76.2 to 86.4% after 1000 cycles. Notably, cells with TEBA^+^ avoided short-circuiting after 2000 cycles, whereas cells with plain ZnSO_4_ electrolyte failed after 1690 cycles, significantly extending operational lifespan. The enhanced electrochemical performance of Zn||VO_2_ cells with TEBA^+^ is attributed to improved reversibility of the Zn anode. TEBA^+^ effectively suppresses parasitic reactions and dendrite growth, ensuring stable cycling and prolonged operational life.

## Conclusions

In conclusion, this study successfully developed a (002)-textured Zn anode with an ultra-high relative texture coefficient (RTC) of 99.8% via a current controlled electrodeposition process, addressing the limitations of standard ZnSO_4_ electrolytes in maintaining (002) orientation—a critical factor for preventing cell failure. The incorporation of TEBAC into the electrolyte not only preserved the (002) texture but also significantly enhanced Zn anode stability by promoting uniform Zn^2+^ deposition and suppressing dendrite growth through selective TEBA^+^ cation adsorption on the Zn surface, which resulting in in-situ conversion of commercial Zn into highly crystalline (002)-textured Zn. Furthermore, the replacement of Cl^−^ with SO_4_^2−^ in TEBAC effectively eliminated pitting corrosion caused by chloride ions. These synergistic mechanisms delivered exceptional performance, including a cycle life exceeding 9000 h at a current density of 1 mA cm^−2^ with a capacity of 1 mAh cm^−2^ in Zn||Zn cells and a coulombic efficiency of 99.7% sustained over 1000 cycles in Zn||Cu cells. Additionally, the Zn||VO₂ full cell demonstrated remarkable stability, achieving 86.4% capacity retention after 1000 cycles at a high current density of 10 A g⁻^1^. These findings highlight the critical role of the (002) texture in enhancing Zn anode performance and underscore the indispensable contribution of TEBA^+^ in achieving this advancement. This work provides a significant step toward the development of next-generation aqueous Zn metal batteries with improved durability and efficiency.

## Supplementary Information

Below is the link to the electronic supplementary material.

Supplementary file1 (DOCX 12686 KB)
