# Supplementary material for: Breaking Performance Limits of Zn Anodes in Aqueous Batteries by Tailoring Anion and Cation Additives
Source: Nanomicro Lett. 2025 May 19;17:259. doi: 10.1007/s40820-025-01773-6 (PMC12089595; doi:10.1007/s40820-025-01773-6)
Supplement: Supplementary file 1 — Supplementary file1 (DOCX 12686 KB) [file 40820_2025_1773_MOESM1_ESM.docx]

Supporting Information for

**Breaking Performance Limits of Zn Anodes in Aqueous Batteries by Tailoring Anion and Cation Additives**

Zhaoxu Mai^1^, Yuexing Lin^1^, Jingying Sun^3^, Chenhui Wang^1^, Gongzheng Yang^1^ *, Chengxin Wang^1, 2^ *

^1^ School of Materials Science and Engineering, Sun Yat-sen (Zhongshan) University, Guangzhou 510275, P. R. China

^2^ State Key Laboratory of Optoelectronic Materials and Technologies, Sun Yat-sen (Zhongshan) University, Guangzhou 510275, P. R. China

^3^ Instrumental Analysis and Research Center, Sun Yat-sen (Zhongshan) University, Guangzhou 510275, P. R. China

*Corresponding authors. E-mail: [wchengx@mail.sysu.edu.cn](mailto:wchengx@mail.sysu.edu.cn) (Chengxin Wang); [yanggzh5@mail.sysu.edu.cn](mailto:yanggzh5@mail.sysu.edu.cn) (Gongzheng Yang)

**Supplementary Figures**


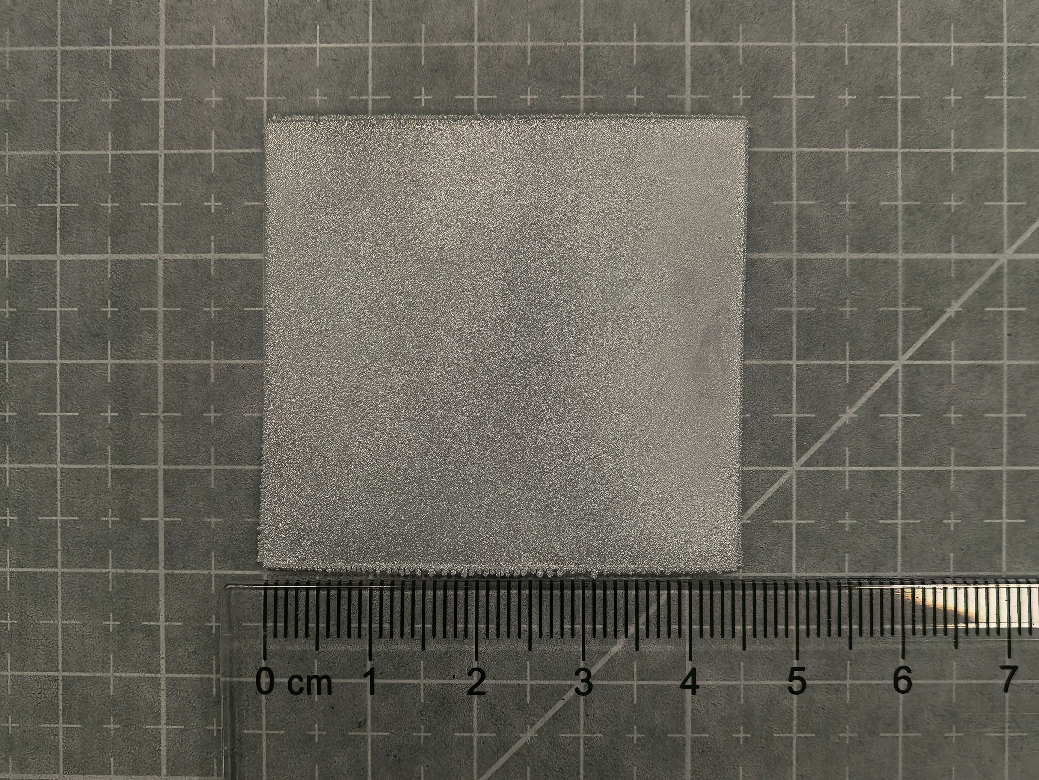


**Fig. S1** Optical images of electrodeposition Zn(002) electrode


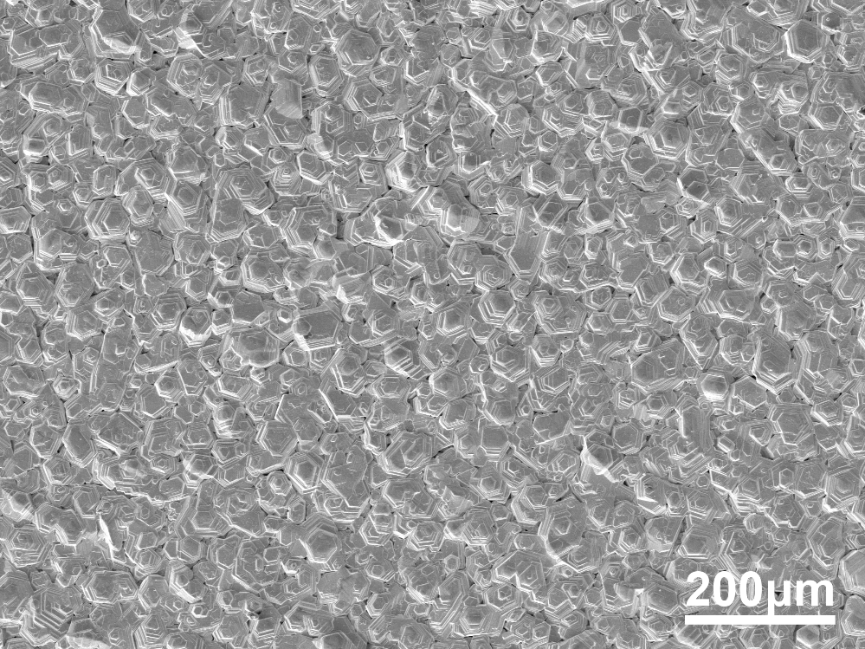


**Fig. S2** SEM images of electrodeposition Zn(002) electrode


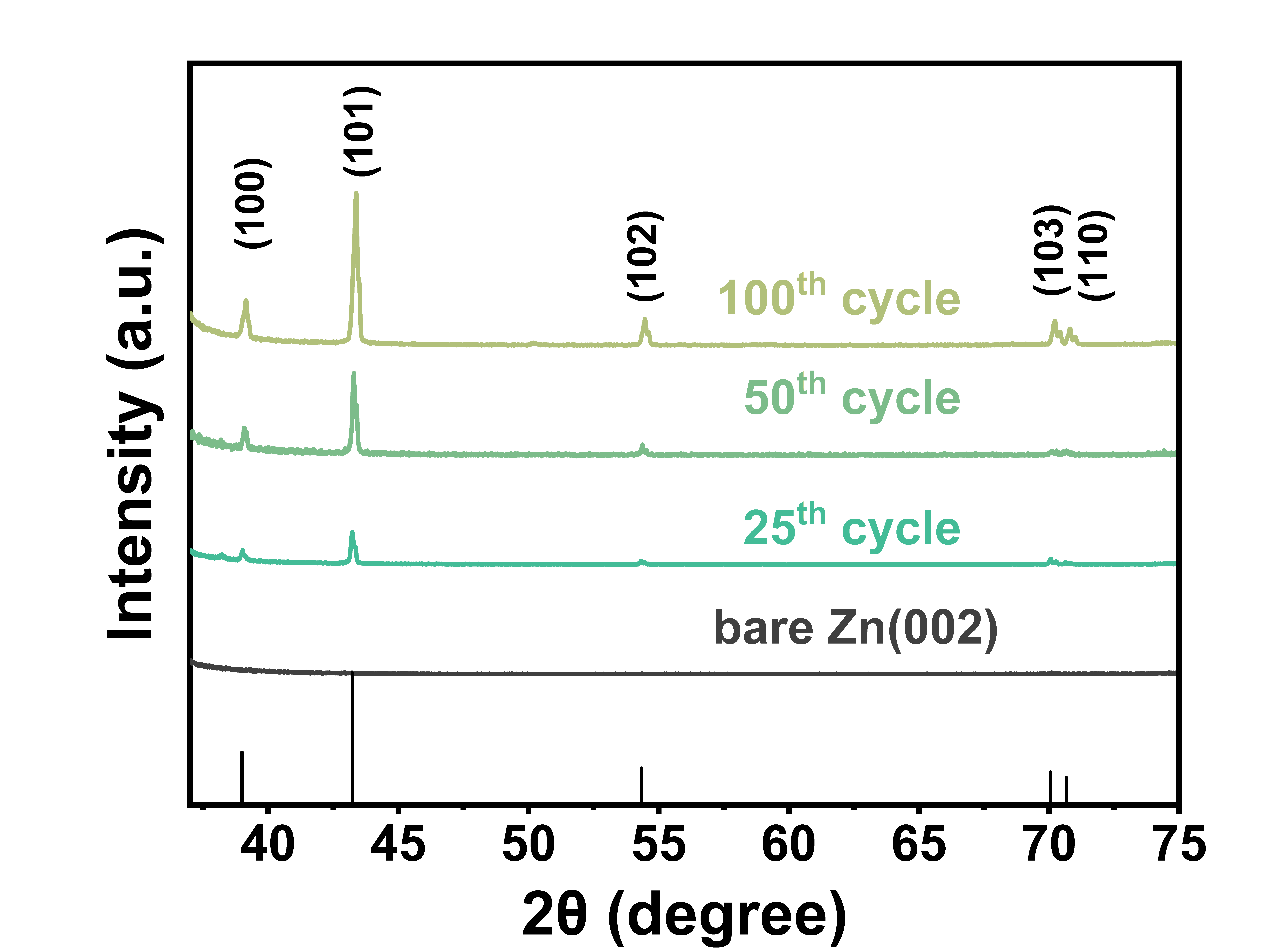


**Fig. S3** XRD patterns of Zn(002) anodes in 2M ZnSO_4_ electrolyte at different cycle stages


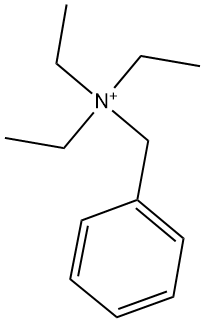


**Fig. S4** The molecule structure of TEBA^+^ ion


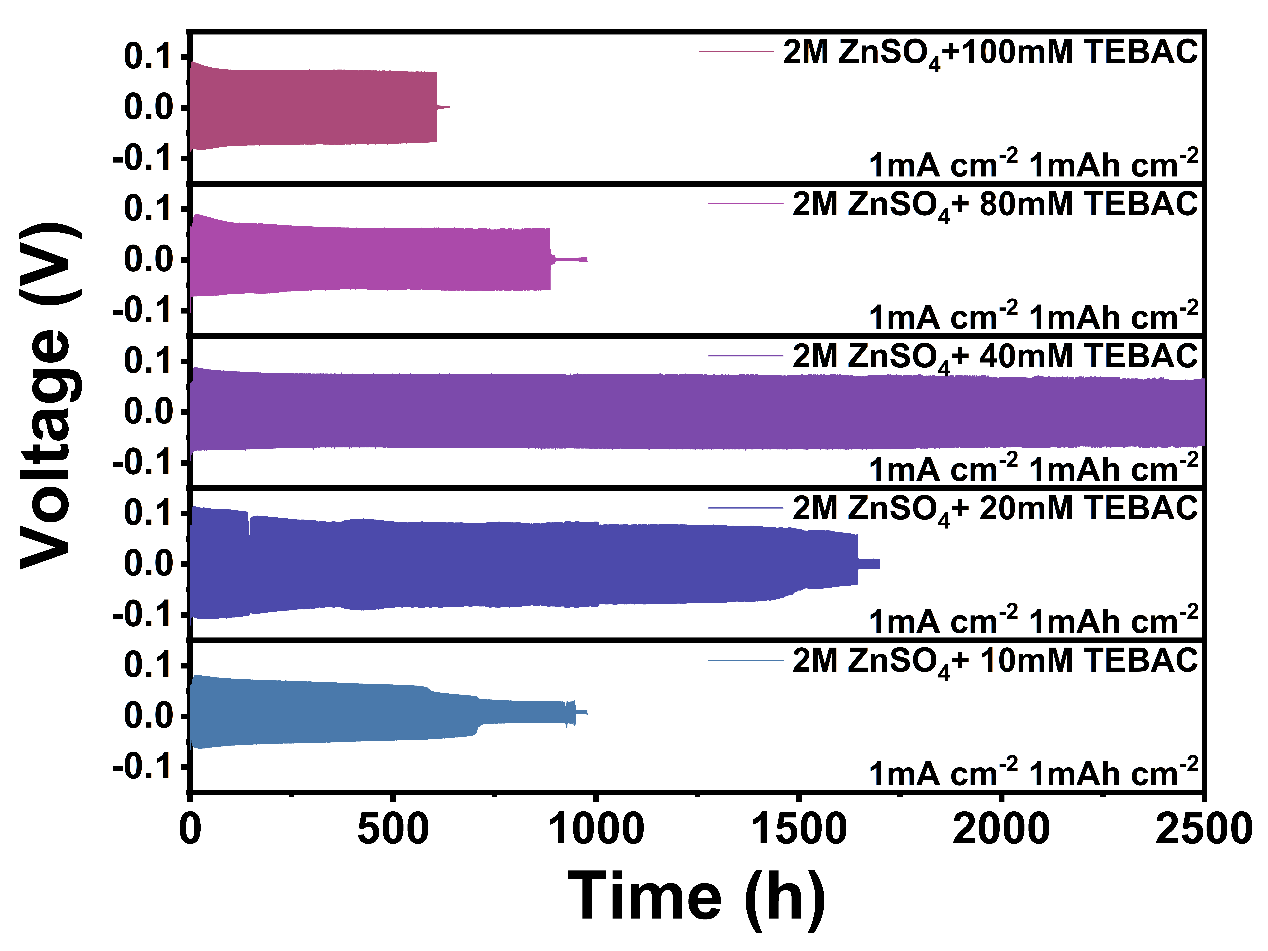


**Fig. S5** Voltage profiles of Zn||Zn symmetric cells with different concentration of TEBAC


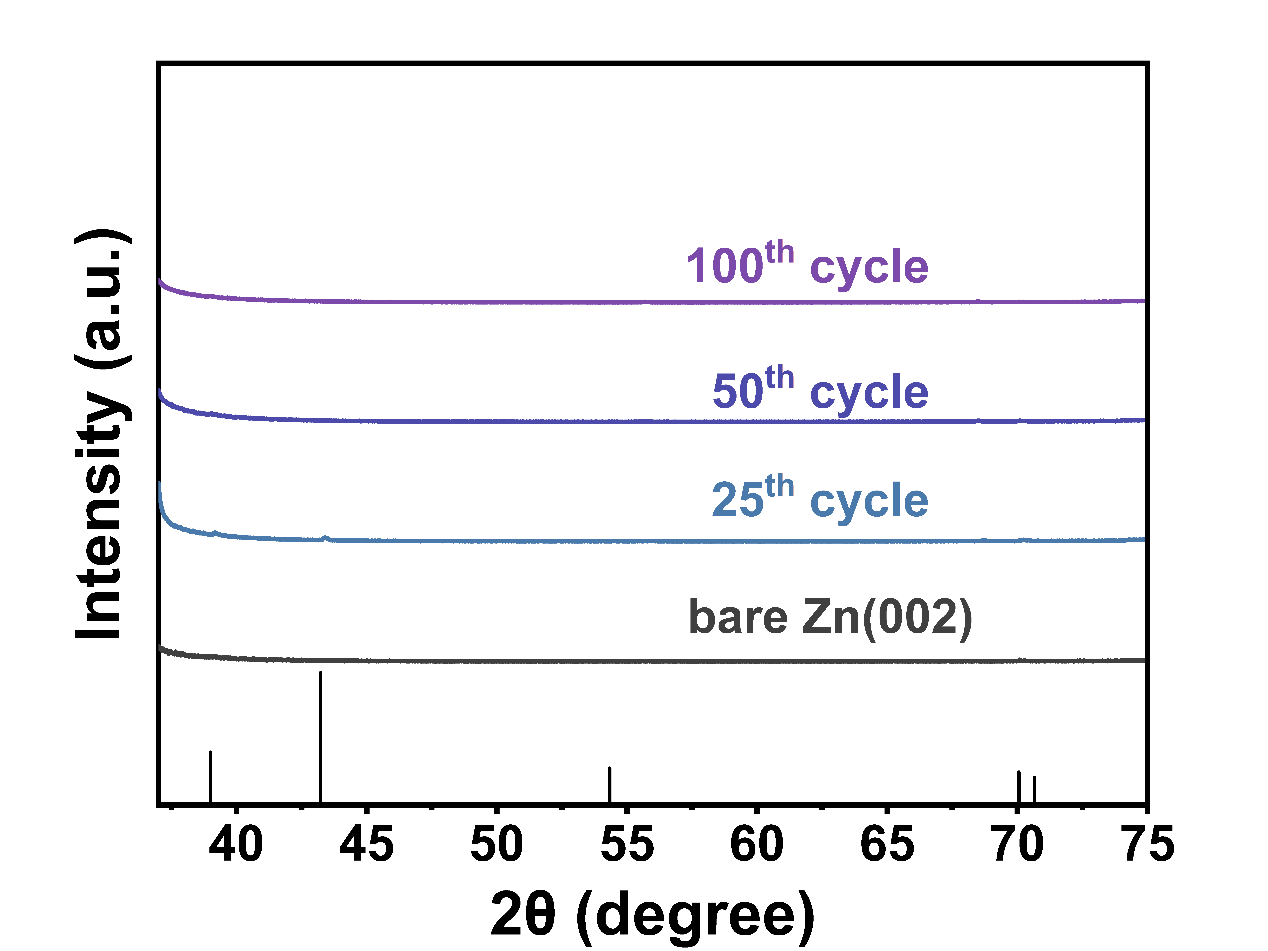


**Fig. S6** XRD patterns of Zn(002) anodes in 2M ZnSO_4_ + 0.04M TEBAC electrolyte at different cycle stages


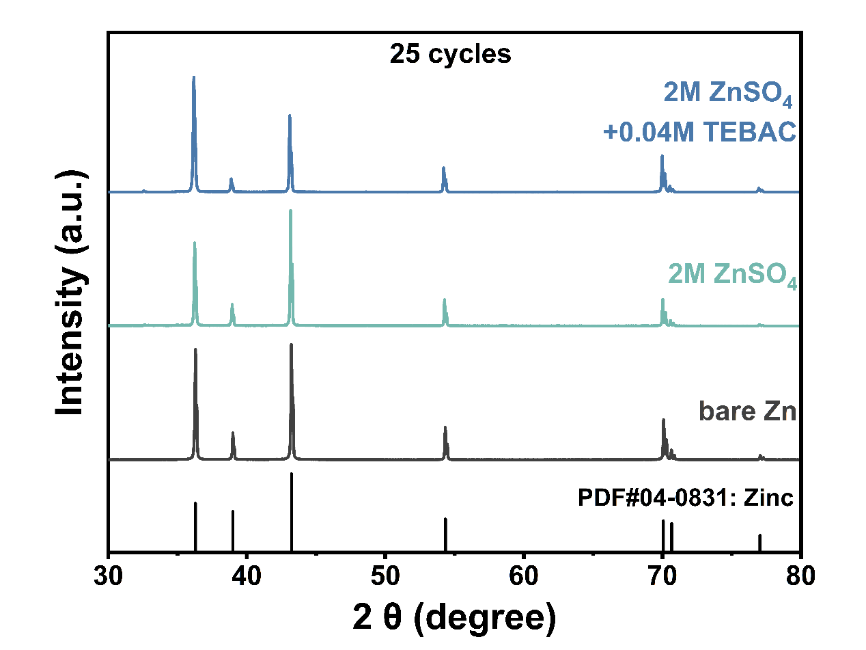


**Fig. S7** XRD patterns of Zn anodes cycled in different electrolytes


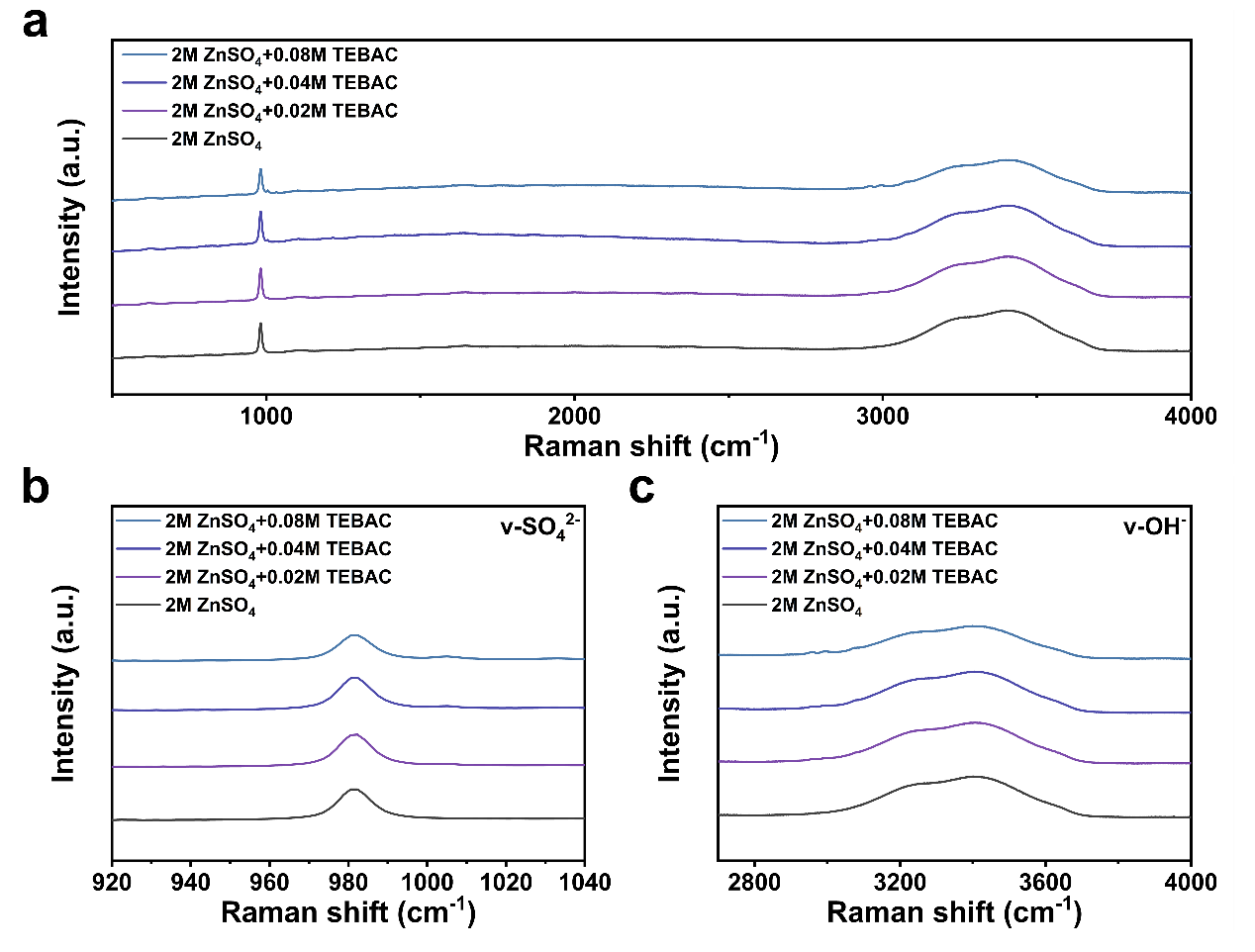


**Fig. S8** Raman spectra of ZnSO_4_ electrolytes with different concentrations of TEBAC


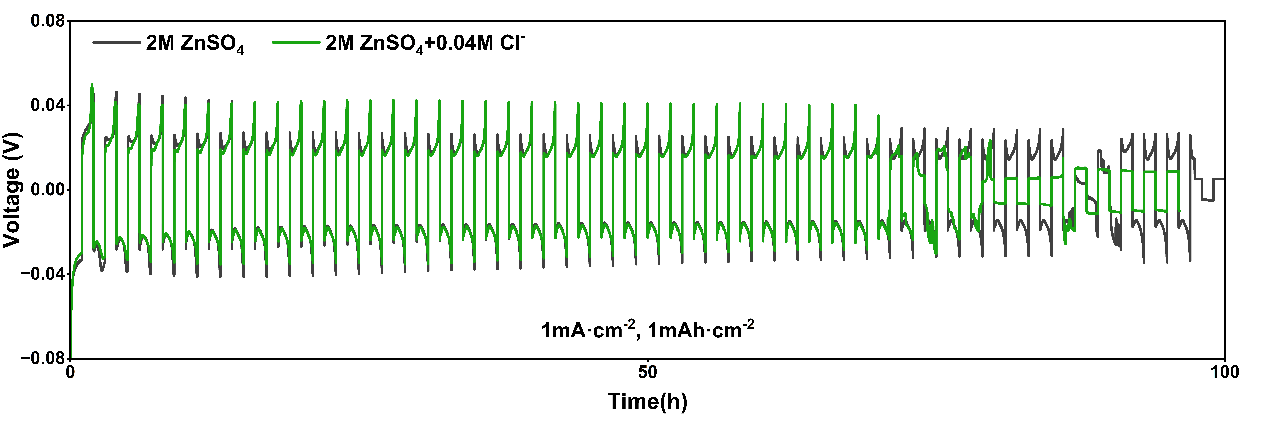


**Fig. S9** Zn||Zn cells cycled in 2M ZnSO_4_ and 2M ZnSO_4_+0.02M ZnCl_2_ electrolyte, respectively


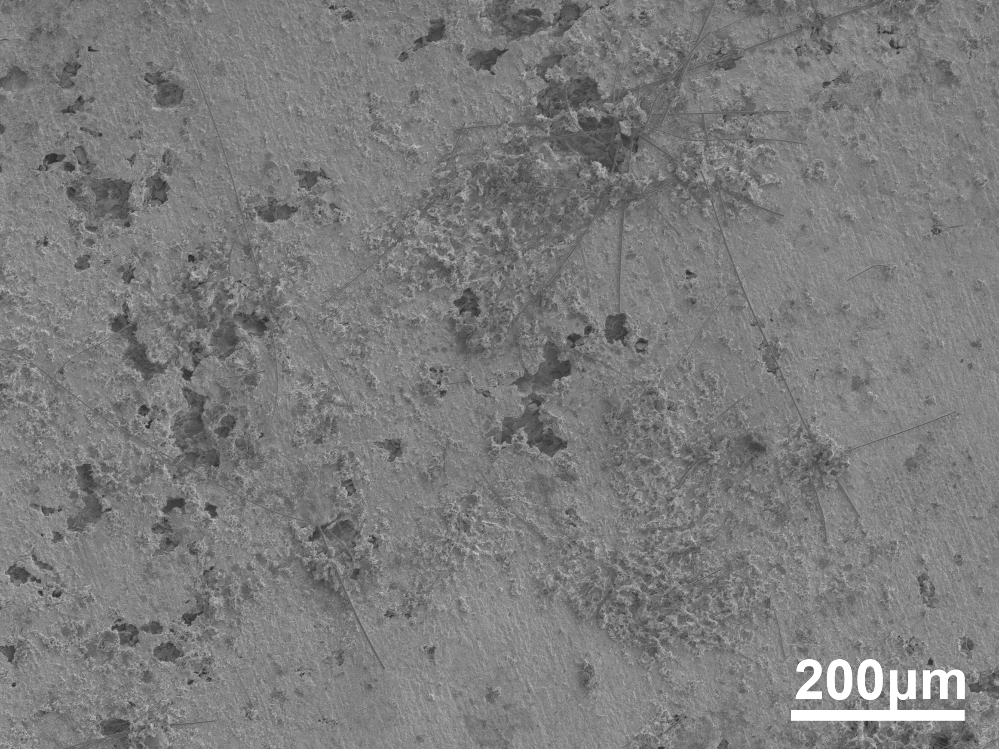


**Fig. S10** SEM images of Zn anodes cycled in 2M ZnSO_4_+0.02M ZnCl_2_ electrolyte


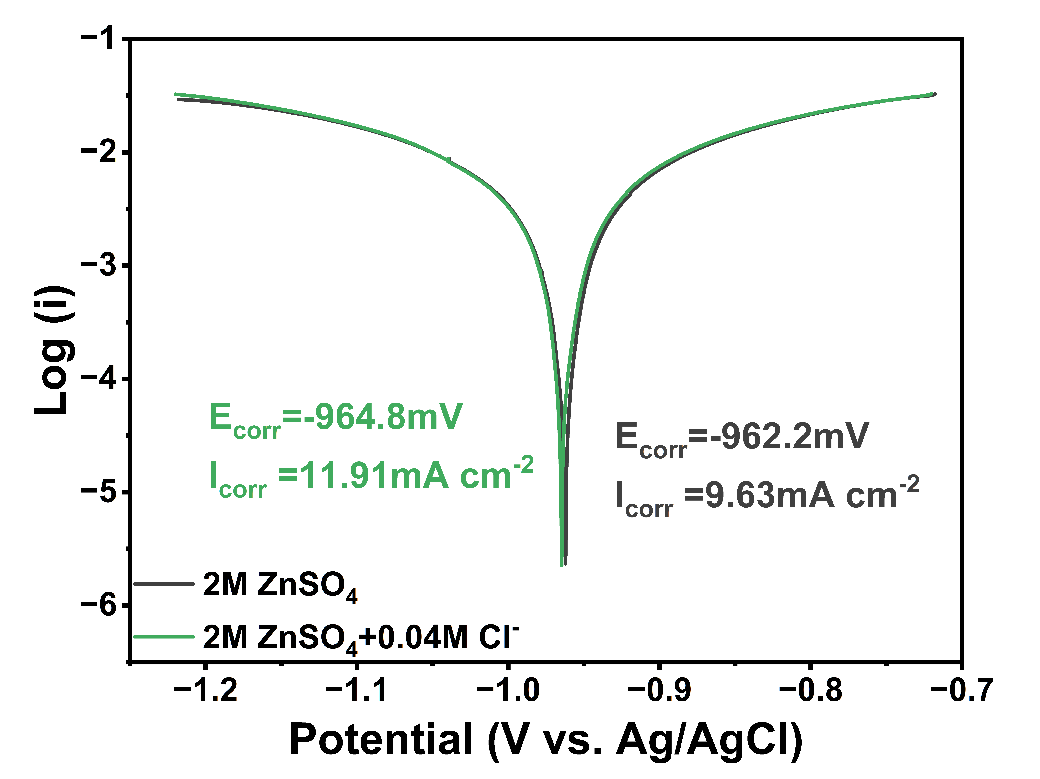


**Fig. S11** Tafel plot of 2M ZnSO_4_ and 2M ZnSO4+0.02M ZnCl_2_ electrolyte


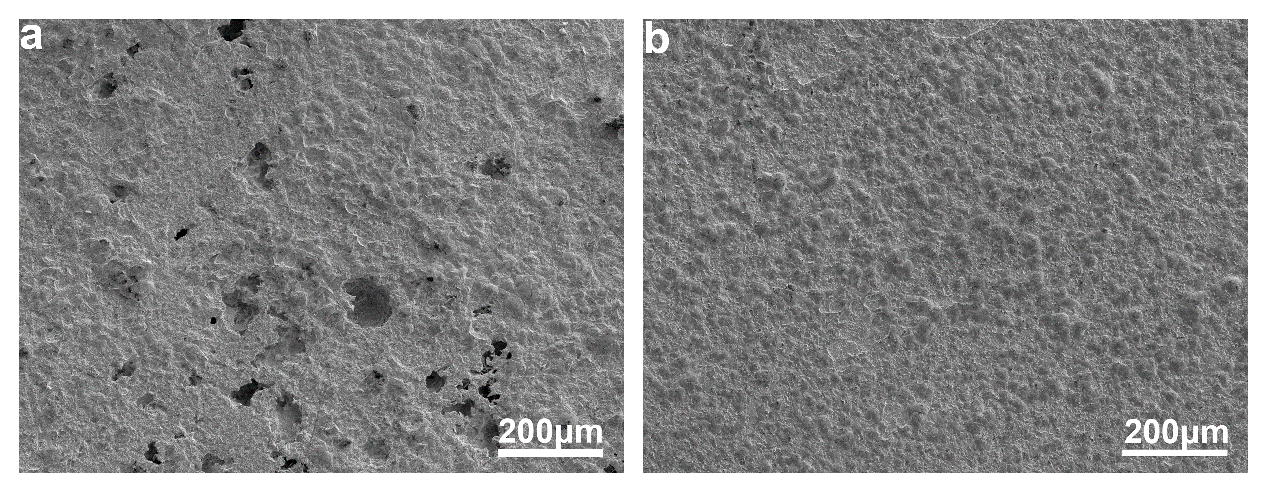


**Fig. S12** SEM images of Zn anodes cycled in : a) 2M ZnSO_4_+0.04M TEBAC electrolyte; b) 2M ZnSO_4_+0.04M TEBA^+^. (50 cycles, after palting)


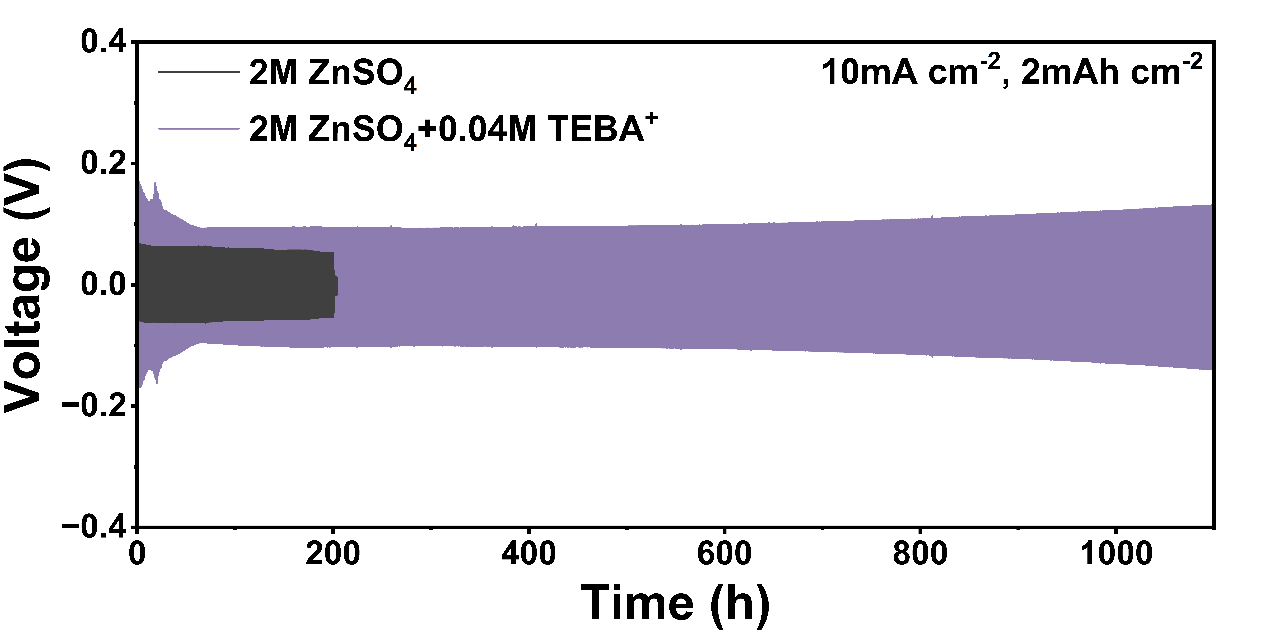


**Fig. S13** Zn||Zn cells cycled in 2M ZnSO_4_ and 2M ZnSO_4_+0.04M TEBA^+^ electrolyte, respectively


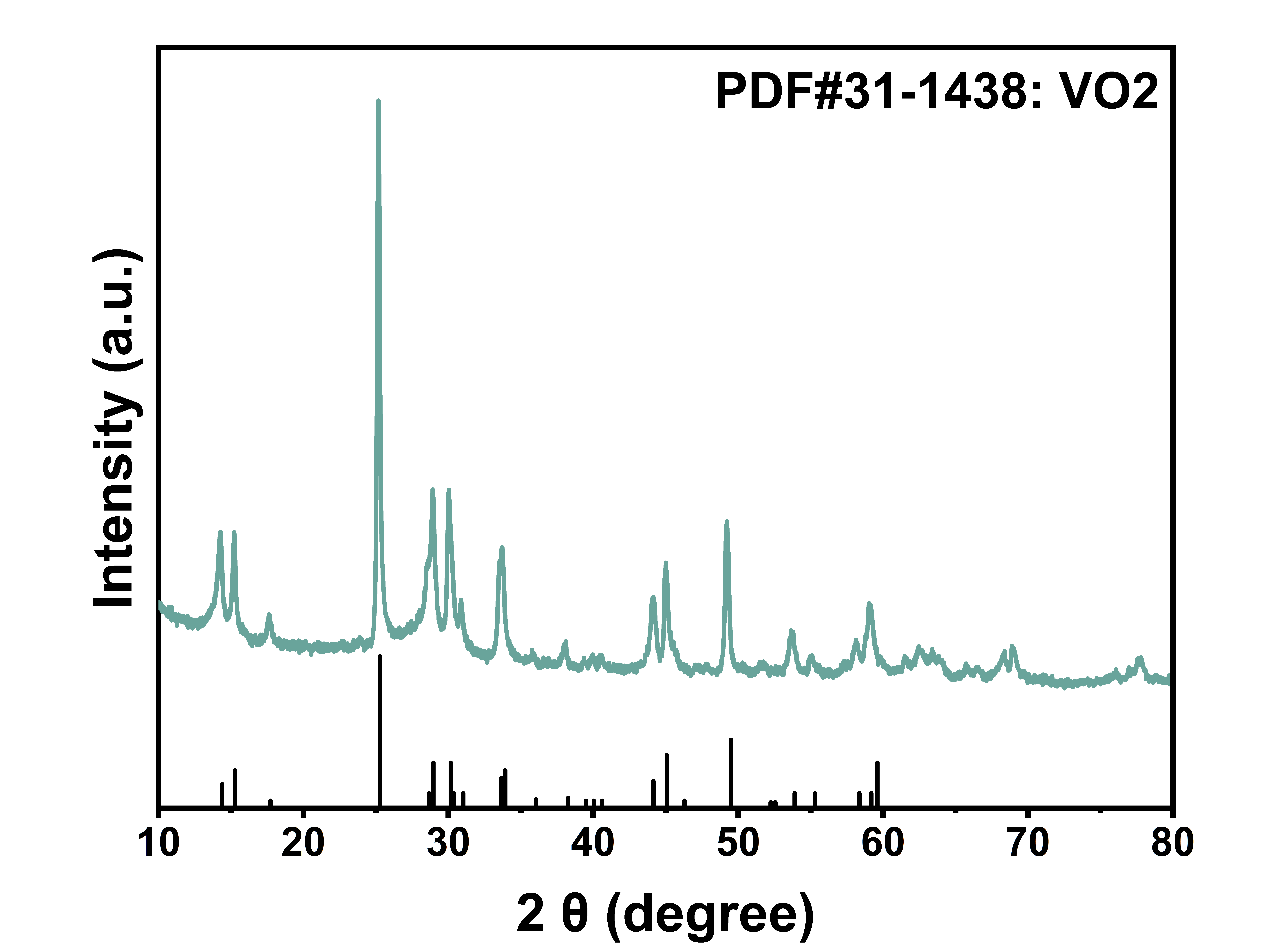


**Fig. S14** XRD pattern of VO_2_ powder


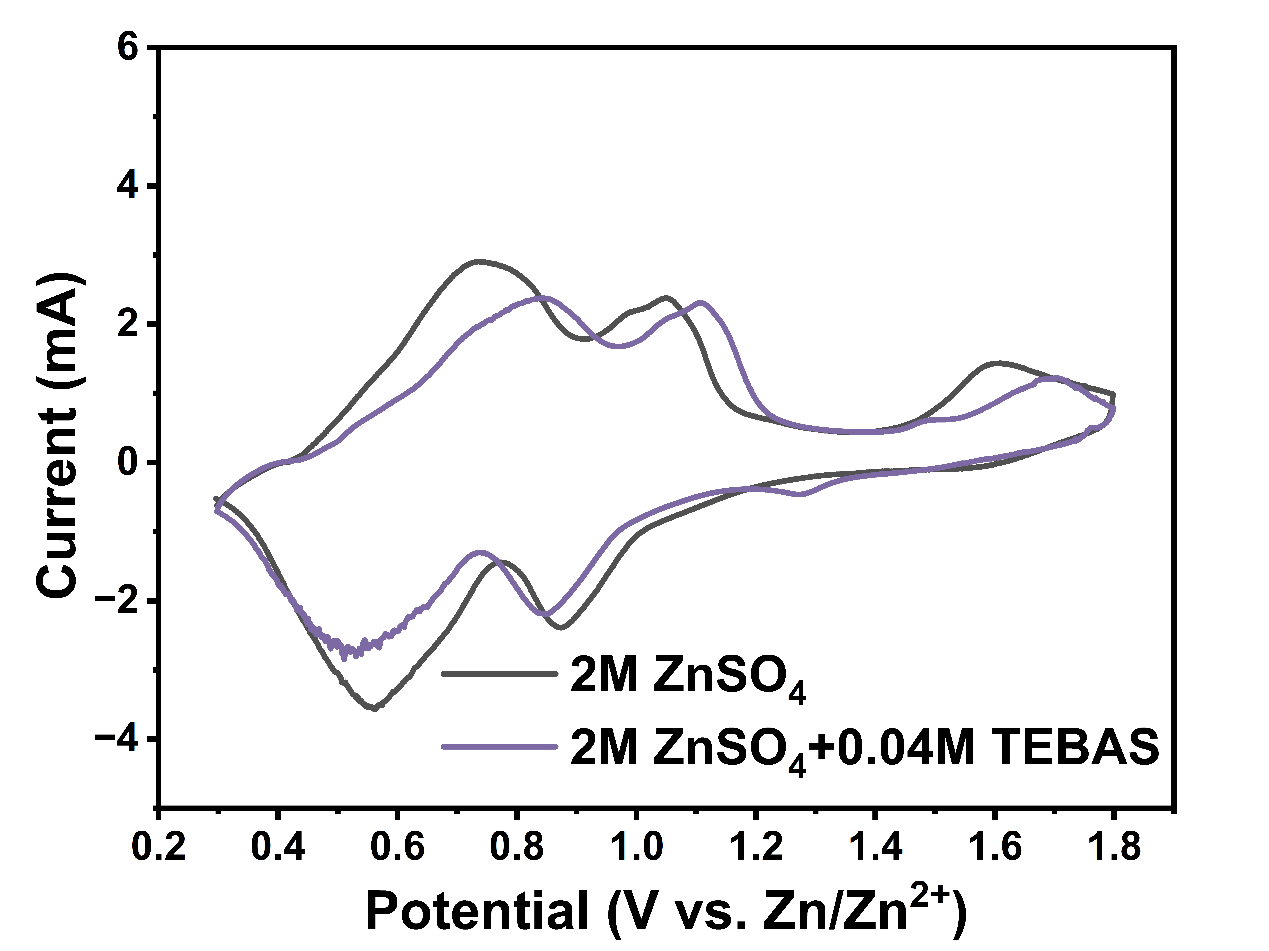


**Fig. S15** The CV profiles of Zn||VO_2_ full cells in different electrolytes at 1 mV s^−1^


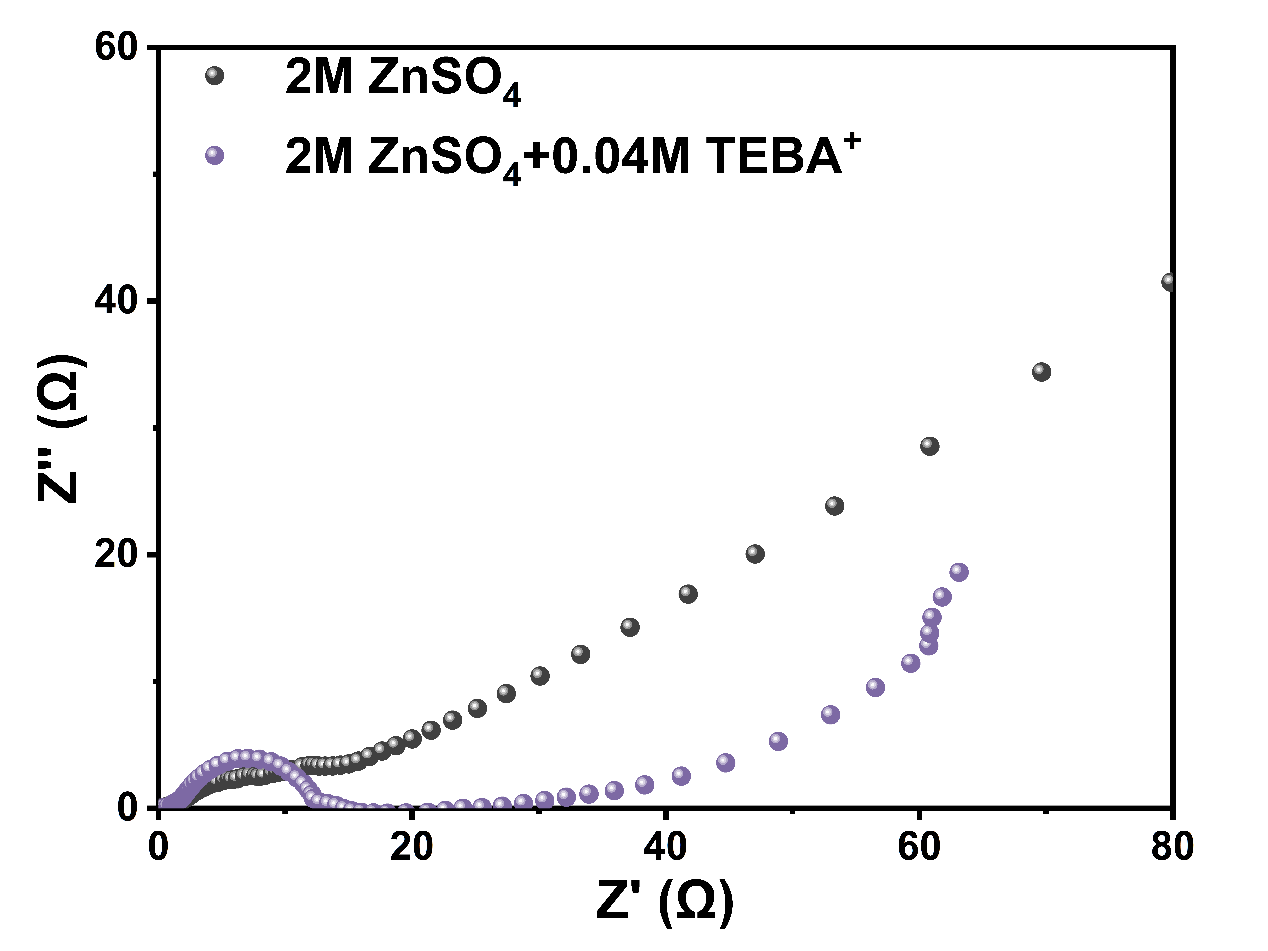


**Fig. S16** EIS spectra of Zn||VO_2_ cells at the pristine state


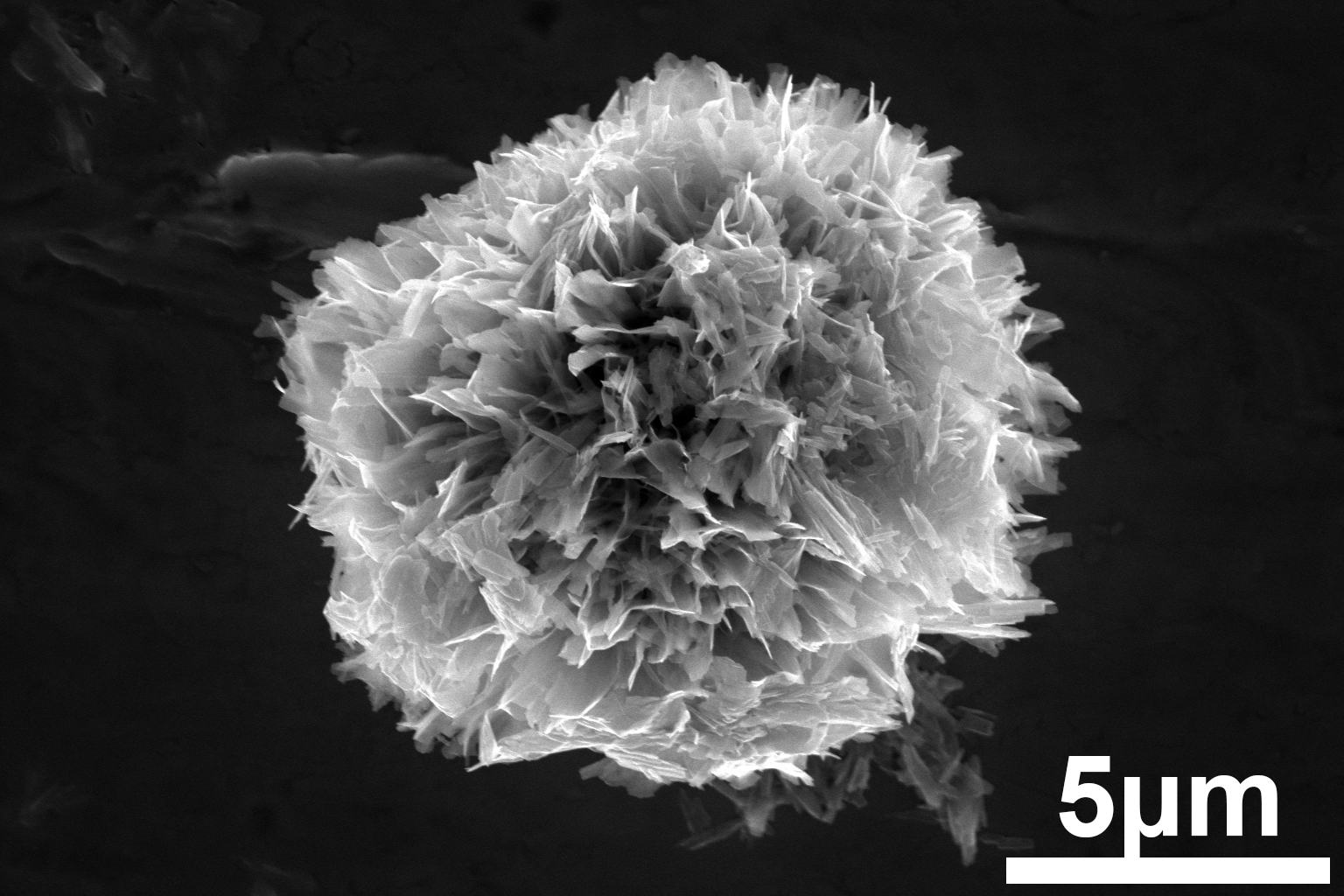


**Fig. S17** SEM image of VO_2_ cathode materials


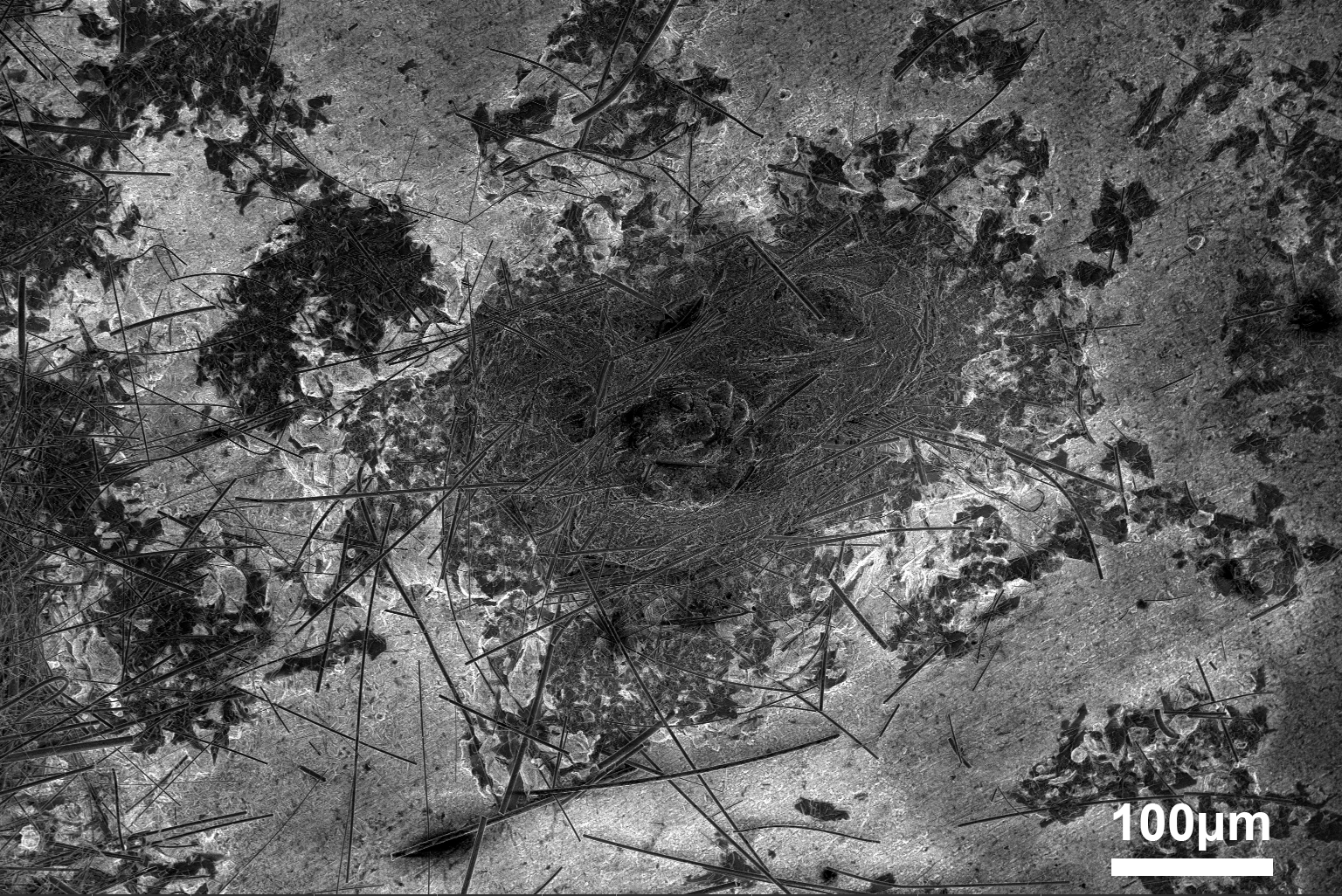


**Fig. S18** SEM image of Zn anode in Zn||VO_2_ full cells tested in 2M ZnSO_4_ electrolyte for 100 cycles at 1A g^-1^ (showing dendrite formation)


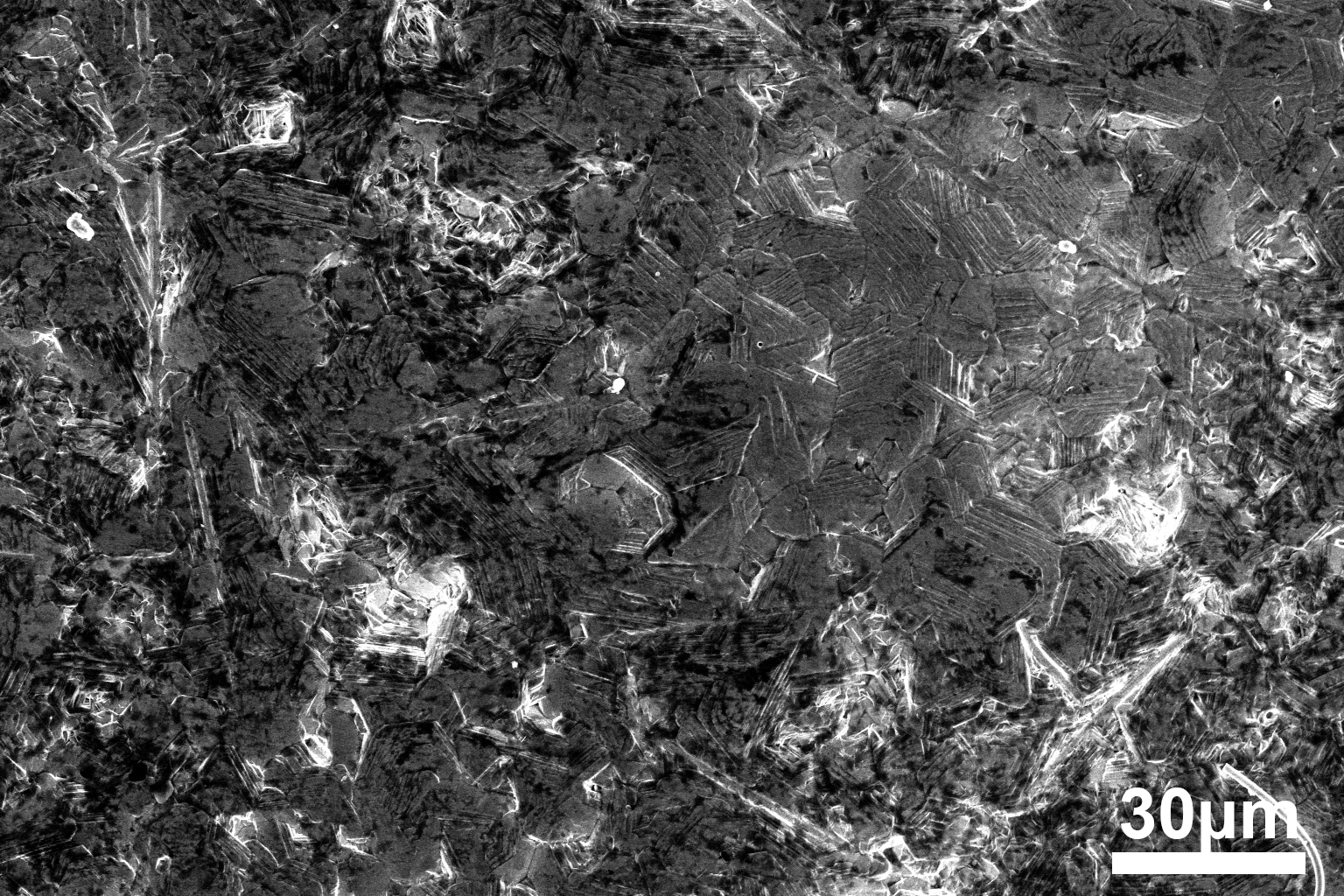


**Fig. S19** SEM image of Zn anode in Zn||VO_2_ full cells tested in 2M ZnSO_4_ +0.04M TEBA^+^ electrolyte for 100 cycles at 1A g^-1^ (showing highly (002)-textured surface without dendrite formation)


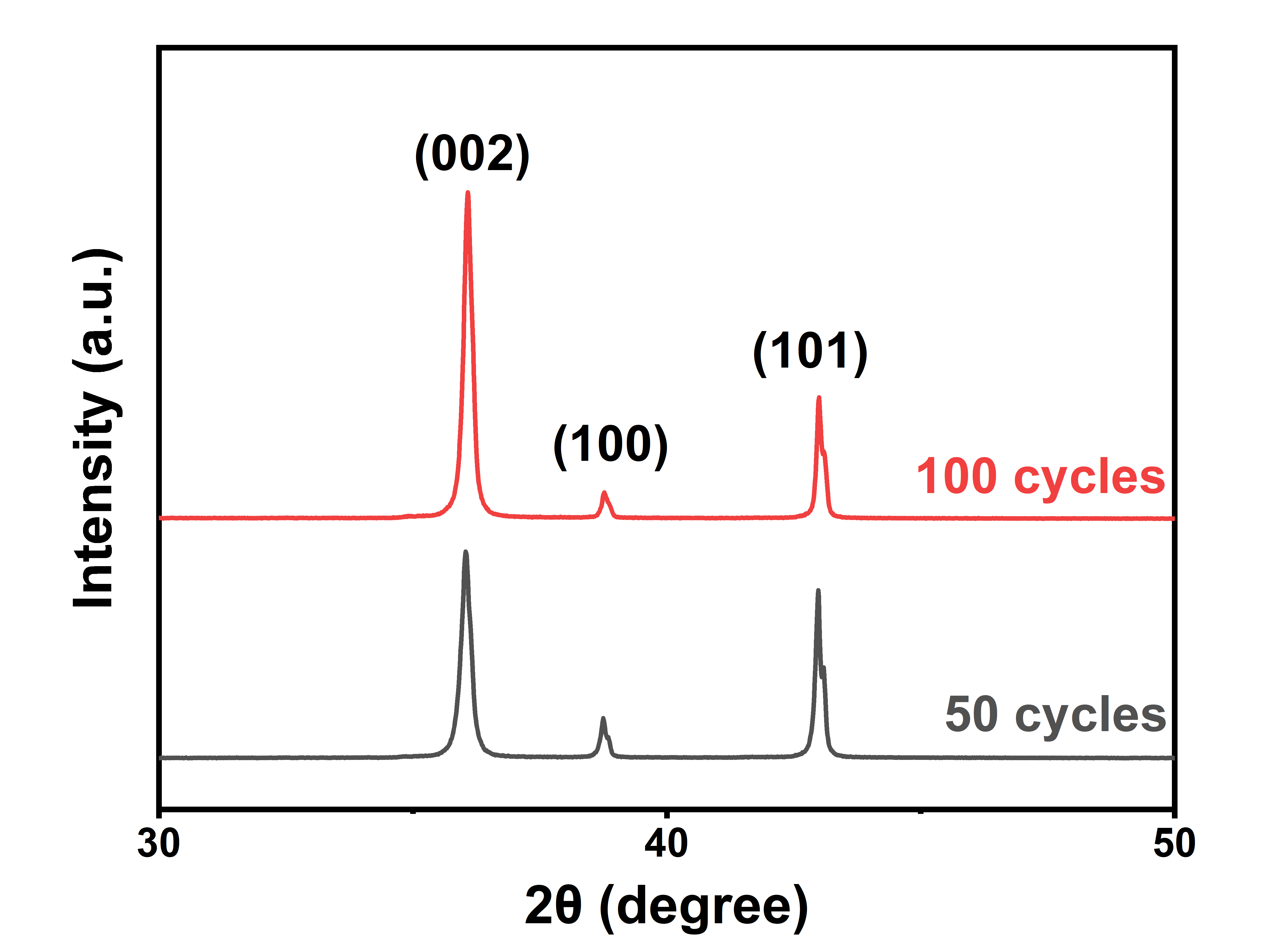


**Fig. S20** XRD patterns of Zn anode in Zn||VO_2_ full cells tested in 2M ZnSO_4_ +0.04M TEBA^+^ electrolyte for different cycles at 1 A g^-1^


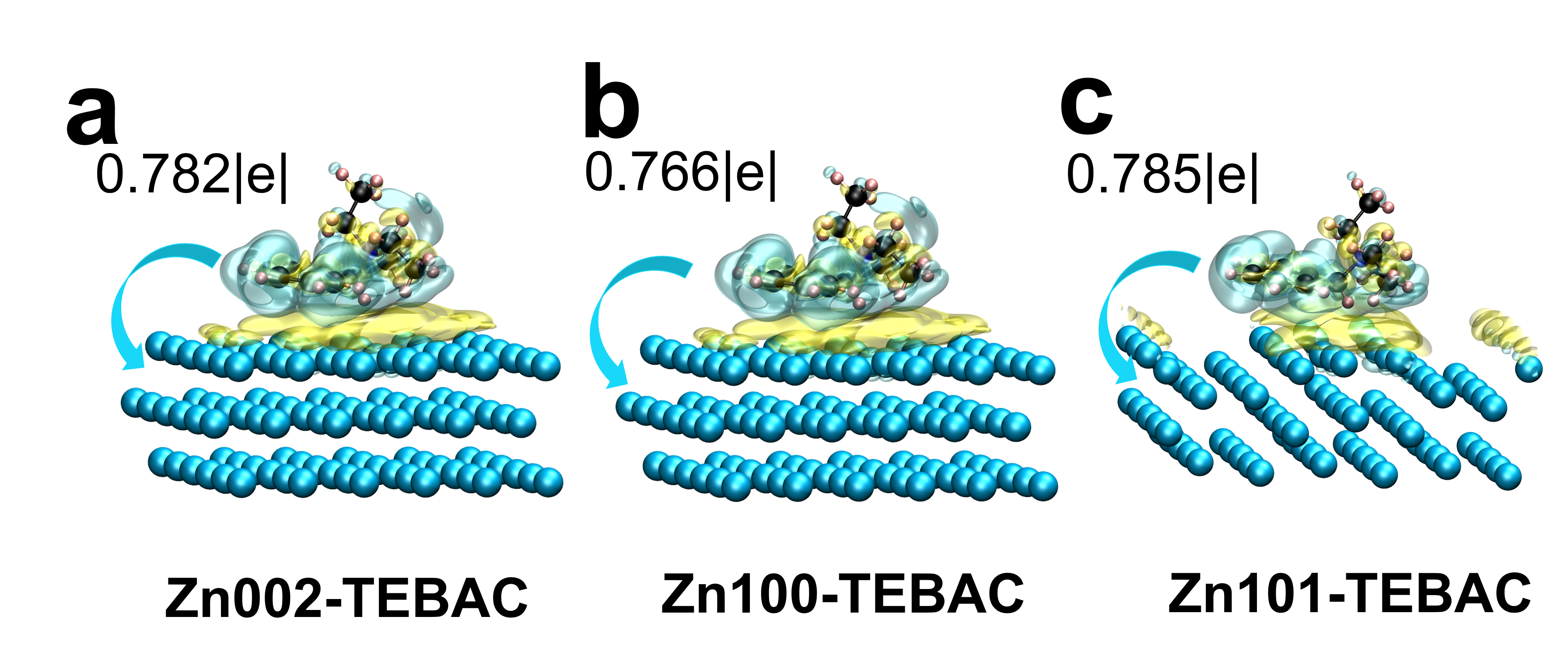


**Fig. S21** The bader analysis for TEBA^+^ over different orientations, the value of which refers to the number of charge transfer from TEBAC to Zn surface. The yellow cloud stands for electron accumulation, and the cyan means electron loss. The adsorption conducted large amount of electron redistributed over TEBAC molecule and enough accumulation between molecule and Zn surface to ensure strong adsorption of TEBAC over the 002, 100, and 101 surfaces, which is mainly transferred from carbon chain

**Fig. S22** The surface energy analysis for different orientated bare Zn surface
